# Unprecedented Biodegradable Cellulose-Derived Polyesters with Pendant Citronellol Moieties: From Monomer Synthesis to Enzymatic Degradation

**DOI:** 10.3390/molecules26247672

**Published:** 2021-12-18

**Authors:** Aihemaiti Kayishaer, Sami Fadlallah, Louis M. M. Mouterde, Aurélien A. M. Peru, Yasmine Werghi, Fanny Brunois, Quentin Carboué, Michel Lopez, Florent Allais

**Affiliations:** URD Agro-Biotechnologies Industrielles (ABI), CEBB, AgroParisTech, 51110 Pomacle, France; aihemaiti.kayishaer@gmail.com (A.K.); aurelien.peru@agroparistech.fr (A.A.M.P.); yasmine.werghi1@gmail.com (Y.W.); fanny.brunois@agroparistech.fr (F.B.); quentin.carboue@agroparistech.fr (Q.C.); michel.lopez@agroparistech.fr (M.L.)

**Keywords:** Levoglucosenone, oxa-Michael addition, Baeyer-Villiger oxidation, branched polymers, renewable polyesters, sustainability, biodegradation

## Abstract

Levoglucosenone (LGO) is a cellulose-derived molecule that is present commercially on a multi-ton/year scale. Taking advantage of the α,β-conjugated ketone of LGO, a new citronellol-containing 5-membered lactone (HBO-citro) was synthesized through a one-pot two-step pathway involving oxa-Michael addition and Baeyer-Villiger oxidation. The solvent-free treatment of HBO-citro with NaBH_4_ at room temperature led to the full reduction of the lactone moiety which gave a novel fully renewable triol monomer having a citronellol side chain (Triol-citro). Noticeably, by simply changing the reducing agent, temperature and reaction duration, the partial reduction of HBO-citro can be achieved to yield a mixture of 5- and 6-membered Lactol-citro molecules. Triol-citro was chosen to prepare functional renewable polyesters having citronellol pendant chains via polycondensation reactions with diacyl chlorides having different chain lengths. Good thermal stability (*T*_d5%_ up to 170 °C) and low glass transition temperatures (as low as −42 °C) were registered for the polyesters obtained. The polymers were then hydrolyzed using a commercial lipase from *Thermomyces lanuginosus* (Lipopan^®^ 50 BG) to assess their biodegradability. A higher degradation profile was found for the polyesters prepared using co-monomers (acyl chlorides) having longer chain lengths. This is likely due to the decreased steric hindrance around the ester bonds which allowed enhanced accessibility of the enzyme.

## 1. Introduction

**Figure 1:**
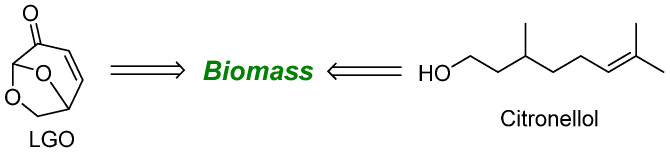
Structural representation of the biomass-derived levoglucosenone (LGO) and citronellol molecules.

Thinking green is crucial not only to resolve the environmental and waste management issues of using polymers [1] but also to turn waste into new high-performing sustainable materials [2]. The current century is witnessing a green revolution where sustainability requirements have overwhelmed almost every polymer industry [3]. Up-to-date, the vast majority of commodity polymers still rely on cheaper but non-renewable fossil feedstocks. To overcome the limited availability of petrochemicals, structural compounds of biomass feedstocks (e.g., lignin, cellulose, terpenes) can be used as abundant and renewable resources to produce chemical building blocks [2]. Levoglucosenone (LGO) is a commercial and renewable chiral molecule that can be obtained at a ton/year scale from cellulosic waste (e.g., sawdust, straw and bagasse) (Figure 1) [4,5]. The great versatility of LGO in diverse synthetic processes [6], such as polymer syntheses [7], has been recently highlighted. Polymers with tailored structures and a wide range of properties, e.g., glass transition (*T*_g_), are now accessible from LGO [7]. For instance, polyacetals with high *T_g_* (up to 100 °C) were prepared through the ring-opening metathesis polymerization (ROMP) of levoglucosenol (the reduced derivative of LGO) [8], and the cationic ring-opening polymerization (CROP) of the methylated analog of levoglucosenol [9]. Highly thermostable functional polymers with *T*_d5%_ up to 401 °C and *T*_g_ of −16.8 °C were also prepared through the ROMP of norbornene-containing LGO-derived monomers [10,11]. Recently, we investigated the preparation of bis(γ-lactone) diol (2H-HBO-HBO) through a MeOH/K_2_CO_3_-mediated Michael addition of LGO followed by H_2_O_2_-mediated Baeyer-Villiger oxidation [12]. 2H-HBO-HBO was found suitable to prepare polyesters that exhibit *T*_g_ values from −1 to 81 °C and good thermostability (142 °C < *T*_d5%_ < 241 °C) [12].

Nonetheless, the use of biomass-derived polymers, such as those from LGO, does not necessarily run over the waste management issues. Indeed, the term “biobased polymers” is sometimes confused with other terms such as “biodegradable polymers” [13]. Biodegradation is the decomposition of material into final metabolic compounds such as CO_2_, through an in vivo or in vitro enzyme-initiated/mediated mechanism [14]. Thus, not all biobased polymers are biodegradable and vice versa. The degradation of a polymer is highly dependent on its characteristics, including optical purity [15], crystallinity [16], molecular weight [17] or chemical structure [18]. From a general point of view, the presence of ester groups along the main chain of the polymer facilitates its enzymatic degradation due to the potential of the ester bonds to undergo hydrolysis [19]. Nevertheless, aromatic-containing polymers such as bio-polyethylene terephthalate (PET) [20], the leading bioplastic in the market, suffer from their resistance to degradation when they are accumulated in the environment.

On the other hand, the performance of the sustainable alternatives remains a crucial parameter to substitute fossil-derived polymers [21]. In this context, functional polymers offer a great potential to modulate the mechanical and thermal properties and enhance the performance of the corresponding polymers [22,23]. Their great reactivity, compared to classical hydrocarbon chains, is due to the presence of pendant and/or terminal functional groups such as hydroxy and C=C moieties. Citronellol is a natural acyclic monoterpenoid found in citronella oil (Figure 1) [24]. It is a highly valuable molecule with anti-microbial and anti-inflammatory properties [25], beneficial for wound healing applications including tissue engineering [26,27]. Allcock et al. took advantage of this molecule to synthesize polyphosphazenes containing citronellol side groups as potential candidates for ligament and tendon tissue engineering [28]. The hydrolysis experiments in deionized water at 37 °C showed a mass loss of 8–16% and a decrease of molecular weight in the range of 28–88% over 12 weeks. Furthermore, they showed that the terminal C=C of citronellol can be crosslinked using ultraviolet (UV). The same authors prepared polyphosphazenes chains containing citronellol esterified amino acids [29]. Although the polymers showed promising properties, their syntheses require polyphosphazenes as the backbone chain to hold the citronellol side group. Regardless of the non-renewability of polyphosphazenes, the latter is synthesized from hexachlorophosphazene that requires chlorine—a dangerous and toxic gas—as a reactant [30]. Breathing chlorine may damage the lungs, in addition, exposure to a low level of chlorine can result in nose, throat and eye irritation [31]. To the best of our knowledge, apart from the aforementioned studies [28,29] the synthesis of citronellol-functionalized monomers and polymers remains scarcely reported.

**Scheme 1:**
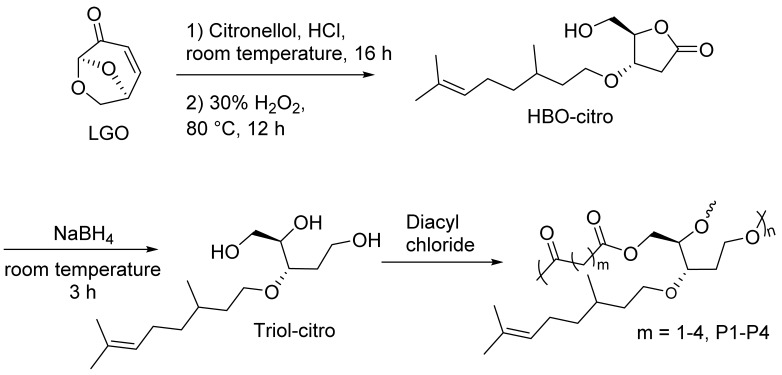
Synthesis of LGO-derived polyesters containing citronellol pendent group.

Since it is highly desirable to develop environmentally-benign monomers and polymers, and considering our strong expertise in LGO, we dedicated ourselves to: (i) functionalize the highly reactive α,β-conjugated double bond of LGO with citronellol through an oxa-Michael addition, (ii) perform our in-house organic solvent-free H_2_O_2_-mediated Baeyer-Villiger oxidation [32] to access HBO-citro, (iii) optimize the reaction conditions to prepare new LGO-derived monomer, and (iv) prepare novel renewable polyesters containing citronellol (Scheme 1). Furthermore, in an attempt to evaluate the biodegradation potential of the prepared polyesters, the enzymatic degradation using Lipopan^®^ 50 BG was also studied.

## 2. Results and Discussion

### 2.1. Synthesis of HBO-Citro

**Scheme 2:**
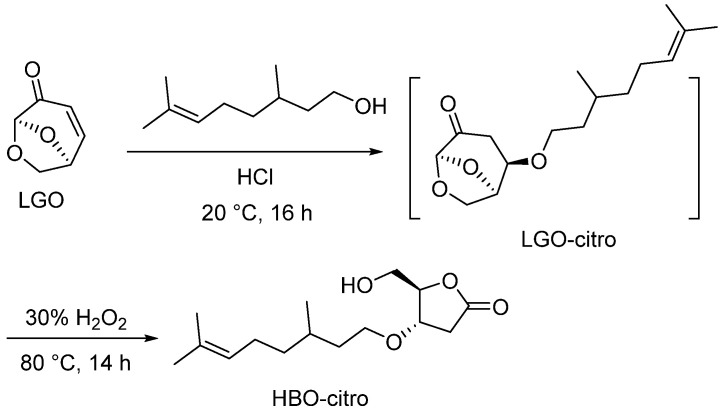
Synthesis of HBO-citro from LGO.

HBO-citro was prepared by a one-pot two-step synthesis from LGO: oxa-Michael addition of citronellol followed by solvent-free Baeyer-Villiger oxidation (Scheme 2). We decided to proceed without isolating LGO-citro not only to minimize the waste generated from the process but also to avoid unnecessary supplementary purification steps which proved to be difficult, i.e., separation of LGO-citro and excess of citronellol. Inspired by the work of Sarotti et al. [33], the oxa-Michael reaction was carried out in an acidic rather than basic medium. Indeed, Sarotti et al. showed that the oxa-Michael addition to LGO and the nature of the resulting product/by-products are highly dependent on the reaction conditions. The optimal outcome was found in an acidic medium using HCl (5 N, 1.5 eq) [33]. Moreover, it is worth mentioning that basic conditions promoted the formation of a dimeric by-product of LGO [33], the dimer LGO-Cyrene^TM^ [12].

We reproduced a similar procedure using HCl (5 N, 1.5 eq) as the acid promoter to activate the Michael acceptor (LGO). An excess of citronellol was employed to drive the reaction towards the formation of LGO-citro. The crude product was then engaged in the H_2_O_2_-mediated Baeyer-Villiger oxidation in absence of organic solvent. Indeed, it is known that the major portion of the discharged waste from a chemical reaction corresponds to the solvents [34]. This necessitates considering solvent-free reactions whenever it is possible. HBO-citro was isolated as a pale-yellow oil in a 58% overall yield. Notably, the aforementioned procedure is scalable up to 60 g without any detrimental effect on the yield or purity of the product. The structure of HBO-citro was confirmed by 1D NMR (^1^H and ^13^C) (Figures S1 and S2, Supporting Information (SI)) and 2D NMR (^1^H-^1^H COSY, ^1^H-^13^C HSQC and ^1^H-^13^C HMBC) (Figures S3–S5, SI), as well as by LC-MS (Figure S25, SI). ^1^H NMR analysis shows the appearance of two AB quartets at 2.81 and 2.44 ppm of the two H_5_ protons (*syn* and *anti*). This was accompanied by the complete disappearance of the conjugated double bond of LGO. Furthermore, H_4_ appears at 3.84 ppm. This considerable shift of H_4_, which normally lies in the range of 2.15–1.96 ppm for (*S*)-γ-hydroxymethyl-γ-butyrolactone (2H-HBO), confirms the successful addition of citronellol. The intact olefin bond of citronellol can be seen at 5.01 ppm (H_13_, ^1^H NMR) and 131.2 and 124.6 ppm (C_14_ and C_13_, ^13^C NMR).

### 2.2. Synthesis of Triol-Citro

**Scheme 3:**
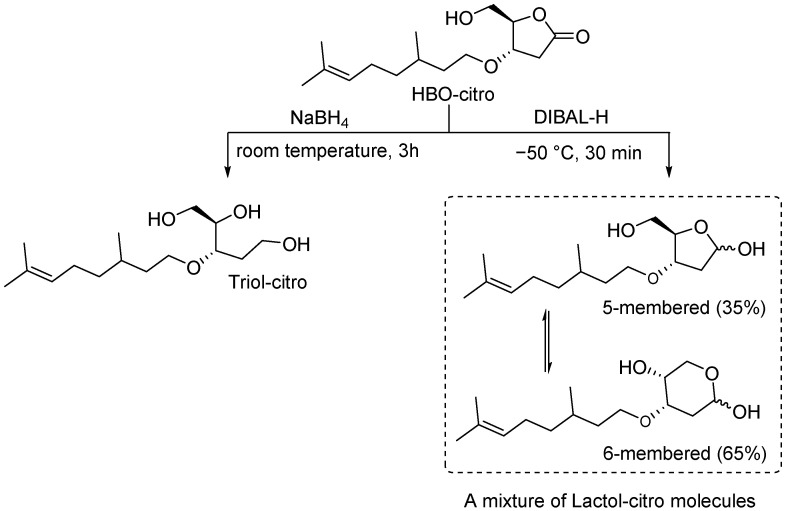
Synthesis of Triol-citro and Lactol-citro from LGO.

Since our goal was to prepare monomers capable of undergoing polycondensation reactions, we then focused our experiments on the reduction of the lactone moiety of HBO-citro (runs 1–11, Table 1). Treatment of HBO-citro in THF at room temperature with 2 eq of NaBH_4_ gave a colorless oil in a 54% yield. NMR analyses of the oily product confirmed the formation of a triol derivative bearing citronellol as a side group (Figures S6–S10, SI) (Scheme 3). The most prominent signals are those of the three hydroxy groups which appear at 4.55, 4.38 and 4.34 ppm (^1^H NMR). Besides, no carbonyl signal belonging to HBO-citro in the region of 170–175 ppm could be detected by ^13^C NMR. Instead, a new carbon peak (C_7_) at 58.2 ppm is observed due to the formation of new hydroxy on this position (see SI for more details). To optimize the reaction yield, the same procedure was performed but under a nitrogen atmosphere leading to an increase in yield to 79%. Letting the reaction run for 3 h increased the yield by 3% to reach 82%. As THF is a non-renewable and toxic solvent, we decided to replace it with a green solvent such as 2-MeTHF, a sugar-derived biofuel. Although proved efficient, this solvent required a longer duration (20 h) to reach a 68% yield. Furthermore, ethanol was tested to conduct the reduction of HBO-citro and resulted in 56% Triol-citro in 3 h. Letting the reaction run for 26 h in ethanol slightly increased the yield to reach 63%.

In an attempt to perform the selective reduction of HBO-citro to synthesize a diol derivative, an alternative route involving diisobutylaluminum hydride (DIBAL-H) as a reducing agent was adopted. More precisely, a 1 M solution of DIBAL-H was added dropwise to a solution of HBO-citro at −50 °C in anhydrous DCM. The reaction mixture was then left to stir at −50 °C for 30 min to afford a colorless oil in 89% yield. The reaction was carried out under a nitrogen atmosphere and strictly anhydrous conditions for a short duration (30 min) to avoid the formation of Triol-citro. The latter was formed in the presence of water traces and for a reaction time exceeding 30 min. Furthermore, if the reaction was performed under air, the reaction yield was dropped to 44%. The performance of 2-MeTHF as a replacement of DCM, a highly toxic molecule, was highlighted (44% yield after a reaction duration of 30 min).

**Figure 2:**
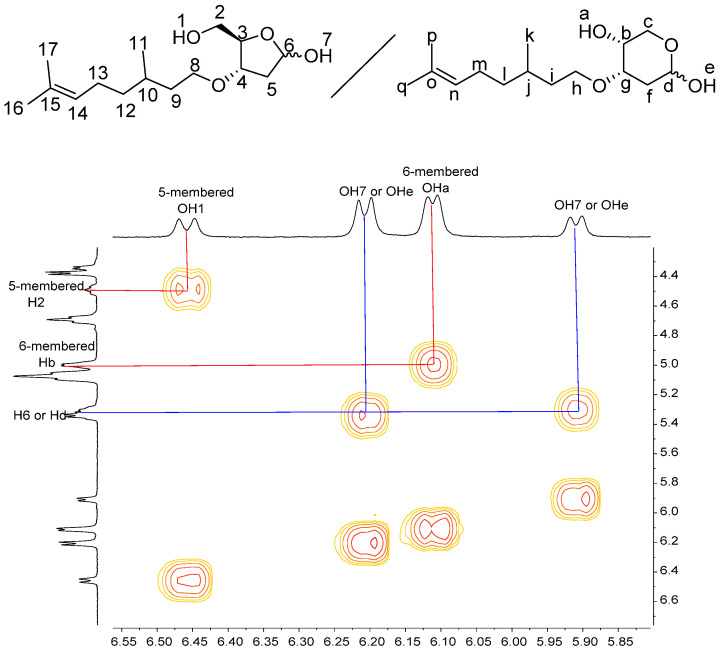
^1^H-^1^H COSY (DMSO-d_6_) spectrum of the Lactol-citro mixture.

Although we were able to avoid the formation of Triol-citro, NMR analyses showed in all cases, the presence of a mixture of 5- and 6-membered cyclic forms of Lactol-citro (Scheme 3). Furthermore, as expected, 2 diastereomers of each cyclic structure are present (See SI, Figures S11–S15, for more details). Indeed, the ring-chain tautomerism of furanoses (5-membered rings) and pyranoses (6-membered rings) is very common in sugars [35]. These furanose-pyranose interconversions exist in equilibrium which led in our case to a mixture of Lactol-citro molecules as depicted in Scheme 3. Although the isolation of one pure product was not possible, ^1^H NMR analysis showed a major occurrence (64%) of the more stable 6-membered derivative. Indeed, the presence, as well as the (inter-)conversions, of both rings can be monitored thanks to the proton resonances of the 4 OH signals at 6.46, 6.21, 6.11 and 5.91 ppm. More precisely, as shown in Figure 2, careful examination of the ^1^H-^1^H COSY spectrum led to the attribution of the peaks at 6.26 and 6.11 ppm to the OH groups of the 5- and 6-membered lactol rings, respectively.

### 2.3. Polycondesation

Having optimized the straightforward synthesis of Triol-citro, its potential to prepare novel functional renewable polyesters was then investigated. Solvent-free polycondensation reactions were sought. Diacyl chlorides with various chain lengths (m = 1–4) were employed as aliphatic co-monomers (Table 2). As the acyl chlorides are very reactive, the first step of the polycondensation was launched at room temperature for 19 h. The temperature was then increased to 50 °C to allow the formation of polymers with higher molecular weights. The isolated solid polymers were found to be insoluble in all classical solvents (e.g., tetrahydrofuran, dioxane, chloroform), except in dimethylformamide (DMF) and dimethyl sulfoxide (DMSO) where a partial solubility was observed. Nevertheless, such descent solubility was enough to characterize the polymers (vide infra). It is worth mentioning that the above procedure is the optimized one and our trials to synthesize the polyesters by heating at 100 °C, during the first stage or after oligomer formation, led to completely insoluble solid polymers.

As Triol-citro contains three hydroxy groups (two primary and one secondary) with different reactivities, branched/crosslinked polymer structures can be obtained from its polycondensation with the very reactive diacyl chlorides. The assignments of NMR spectra were consistent with the formation of branched polyesters. The most prominent signals are those related to the incorporation of acyl chloride co-monomer accompanied with the disappearance of the free hydroxy groups of Triol-citro. In addition, the olefin signals of citronellol remained intact (see SI for more details). Nevertheless, the limited solubility of the resulting polyesters impeded a full NMR assignment of all peaks/protons. Thus, Fourier-Transform Infra-Red (FTIR) analysis was performed. FTIR spectra of P1-P4 show the disappearance of the characteristics OH band at 3397 cm^−1^ that corresponds to the hydroxy groups of the triol monomer. Furthermore, a band of the carboxyl group of the newly formed ester is displayed at 1730 cm^−1^ (Figure S35, SI). These results confirm the formation of new ester bonds which corresponds to branched polymer chains. Indeed, the condensation reactions with the three hydroxy groups match with the literature reported for other triol molecules (e.g., glycerol) [36]. Taylor and coworkers showed that the formation of linear polyesters from trifunctional glycerol is only possible if diarylborinic acids (Ar_2_BOH) were used [36]. Otherwise, in the absence of a suitable catalyst, crosslinked branched structures were obtained. Unfortunately, the degree of branching (DB)—which is the percentage of dendritic units based on the total number of repeating units—was out of reach in this study. This is due to the low solubility of P1-P4 in all NMR classical solvents which prevented us from clearly determining the relative percentages of the linear and dendritic units within the polymer structures by NMR spectroscopy. In addition, we performed the deconvolution of the peaks to separate the overlapped peaks, however, no valuable information could be retained for the same aforementioned reason.

The molecular weights of the polymers were determined by size exclusion chromatography (SEC). The polyesters obtained from the triol derivative showed small number-average molecular weights (M_n_), e.g., 3.4 kDa for P1 (run 1, Table 2). However, all polymers were very slightly soluble in DMF before SEC analysis and we had some difficulties while filtering the samples due to the formation of crosslinked/branched structures. Thus, the results obtained by SEC only represent the soluble portions of the polymers. We assumed that the reduced solubility can be also due to the presence of long polymer chains. In general, relatively low dispersity (Đ) (1.1–1.6) was registered for P1-P4.

Differential scanning calorimetry (DSC) was used to determine the thermal transitions of P1-P4. Interestingly, negative T_g_ values (−20 °C to −42 °C) were registered for all polymers. Indeed, such low T_g_ values are unprecedented for the family of LGO-derived polymers and that is due to the presence of citronellol side groups. Similar behavior was observed for the polyphosphazenes-citronellol polymers reported by Allcock et al. (T_g_ as low as −87.9) [28,29]. Having T_g_ values in this negative range is also useful for the polymers to be used in biomedical elastomers that necessitate T_g_ lower than body temperature. No melting temperature (T_m_) was observed for any of the polymers reported in this study. Moreover, apart from P4, a drop in T_g_ from −20 °C to −42 °C was observed as the number of carbons in the aliphatic co-monomer unit was increased from 3 (m = 1) to 5 (m = 3). This coincides with what we have reported for the 2H-HBO-HBO-based polyesters [12]. Thermogravimetric analyses (TGA) showed that P1-P4 have good thermal resistance where an effect of the chain length of the acyl chloride was also observed. For example, P4 exhibited T_d5%_ of 170 °C whereas the T_d5%_ of P1 was 142 °C. This difference in thermal stability is even more representative if T_d50%_ is considered, 386 °C for P4 vs. 320 °C for P1.

### 2.4. Enzymatic Degradation

**Figure 3:**
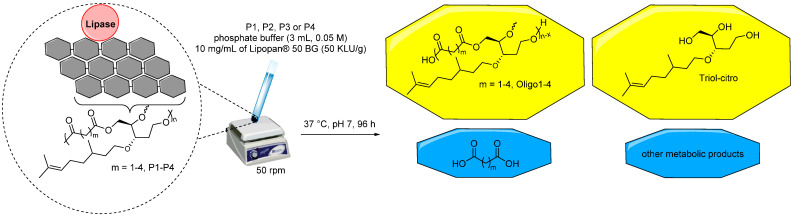
Illustration of the enzymatic degradation of P1-P4 and the corresponding hydrolyzed products.

Evaluating the biodegradation of biobased polyesters is not only of crucial importance to give end products that are less harmful to the environment, but also to select and design biodegradable materials for specific applications such as tissue engineering and drug release. Based on the above promising results from the polycondensation experiments, we investigated the enzymatic degradation of P1-P4 reported in Table 2. An enzyme from Thermomyces lanuginosus (Lipopan^®^ 50 BG; Novozymes^®^, Novozymes AS, Denmark) was chosen to assess biodegradability because it is an industrial enzyme that belongs to the lipase family which is known to hydrolyze the ester bonds (if they are susceptible for degradation) [37]. Indeed, the enzymatic degradation through lipases occurs in two stages: (i) adsorption of the enzymes on the polymer surface which triggers a surface erosion process (especially due to the big size of the enzyme), (ii) hydrolysis of the esters bonds to form the corresponding hydroxy and carboxylic acid terminal oligomers. The aforementioned process as well as the potential products that could be obtained after the enzymatic degradation of P1-P4 are illustrated in Figure 3.

Due to the low solubility of P1-P4, all our trials to make films by solvent casting failed. Thus, we decided to study their biodegradation using their powder form upon grinding at the end of the polymerization reactions. No remarkable degradation could be observed in the case of P1 as judged by the different analytical techniques (vide infra). Indeed, one of the important factors that affect the degradation/hydrolysis of polyesters is the ratio of the constituting aromatic/aliphatic units as well as the length of the aliphatic chains [18]. More precisely, the hydrolytic stage of enzymatic degradation is determined by (i) the hydrolysis time (t_c_) of polymers into shorter chains, and (ii) the water diffusion through the material in this time frame (D_T_) which can be affected by the polymer structure. For instance, a short co-monomer chain such as that of malonyl chloride (m = 1) can induce a more strained globular polymer conformation that hinders the accessibility of the enzyme into the interior surface of the chains and delays/prevents the hydrolysis rate of the ester bonds.

In the case of successful degradation, the hydrolysis of the ester bonds leads to the formation of shorter oligomers with terminal carboxyl acid and hydroxy groups and/or the constituting hydroxy monomer (Figure 3) whose existence can be easily monitored by ^1^H NMR and FTIR. Firstly, no hydroxy chemical shift(s) and vibration band were observed by ^1^H NMR and FTIR, respectively, of the isolated product of P1 degradation experiment. The enzymatic degradation was monitored also using SEC to determine the reduction in M_n_ and changes in Đ. SEC of P1 after degradation showed no influence on the M_n_ (3.4 KDa, Figure S31, SI) (Table 3), moreover, Đ was increased from 1.6 to 2.9. Interestingly, polymers with longer aliphatic chains, e.g., P3, m = 3, showed a drop in M_n_ to reach 0.8 kDa after degradation (Table 3) (Figure S33, SI). In addition, no SEC peak could be detected in the case of P2 and P4 which confirms the absence of long polymer chains in the recovered samples (Figures S32 and S34, respectively, SI). Examination of the ^1^H NMR spectra of P2-P4 after enzymatic degradation, showed the appearance of new peaks, mainly at 4.59 ppm and 12.05 ppm which correspond to the hydroxy and carboxylic acid protons of the hydrolyzed product, respectively (Figure S24, SI). In agreement with the ^1^H NMR, FTIR showed the appearance of a large hydroxy vibration band at around 3350 cm^−1^ after enzymatic degradation (Figure S36, SI). The thermal properties of the resulting short oligomers were also analyzed by DSC where a difference was registered after degradation especially in the case of P3 that showed an increase of T_g_ from −42 °C to −22 °C (ΔT_g_ = 20 °C) (Table 3). Last but not least, the hydrolyzed polymers were weighed after being washed with water (3 × 10 mL), centrifuged and freeze-dried to remove all traces of water. Interestingly, the polymers P2-P4 were degraded at a good rate (∼80% mass loss in 96 h), however, no mass loss could be observed in the case of P1. All these results confirm that P2-P4 were successfully degraded when a lipase such as Lipopan^®^ 50 BG is engaged.

## 3. Materials and Methods

Chemical and reagents. Levoglucosenone was graciously provided by Circa group. Citronellol (Sigma-Aldrich), diisobutylaluminum hydride (DIBAL-H) (Sigma-Aldrich), sodium borohydride >98% (Sigma-Aldrich), malonyl chloride 97% (Sigma Aldrich), succinyl chloride 95% (Sigma Aldrich), adipoyl chloride 98% (Sigma Aldrich), glutaryl chloride 97% (Sigma Aldrich), Lipopan^®^ 50 BG (Novozymes, Danemark) a purified lipase from *Thermomyces lanuginosus* expressed in *Aspergillus oryzae,* were used as received. NMR solvents including CDCl_3_ and DMSO-*d*_6_ were purchased from Cambridge Isotopes Laboratories. HPLC grade solvents were purchased from Thermofisher Scientific and used as received. Ultra-pure laboratory-grade water was obtained from MilliQ, 18.2 megaOhms. TLC analyses were performed on an aluminum strip coated with Silica Gel 60 F254 from Merck, revealed under UV-light (254 nm) then in presence of potassium permanganate staining solution. All manipulations with air-sensitive chemicals and reagents were performed using standard Schlenk techniques on a dual-manifold line, on a high-vacuum line.

Characterization. Nuclear Magnetic Resonance (NMR) spectroscopy. ^1^H NMR spectra were recorded on a Bruker Fourier 300 MHz (CDCl_3_ residual signal at 7.26 ppm and DMSO-*d*_6_ residual signal at 2.5 ppm). ^13^C NMR spectra were recorded on a Bruker Fourier 300 (75 MHz) (CDCl_3_ residual signal at 77.16 ppm and DMSO-*d*_6_ residual signal at 39.52 ppm). Data are reported as follows: chemical shift (δ ppm), assignment. All NMR assignments were also made using ^1^H-^1^H COSY, ^1^H-^13^C HMBC and ^1^H-^13^C HSQC spectra.

Size exclusion chromatography (SEC) was performed at 50 °C using an Agilent Technologies 1260 Infinity Series liquid chromatography system with an internal differential refractive index detector, a viscometer detector, a laser and two PLgel columns (5 µm MIXED-D 300 × 7.5 mm) using 10 mM Lithium Bromide in HPLC grade dimethylformamide as the mobile phase at a flow rate of 1.0 mL/min. Calibration was performed with poly(methyl methacrylate) standards from Agilent Technologies. Typically, ~3 mg of each sample was dissolved in 1 mL of DMF (10 mM LiBr) prior to analysis.

Thermogravimetric Analysis (TGA) was measured with a TGA Q500 (TA Instruments). Typically, ~2 mg of each sample was equilibrated at 50 °C for 30 min and was flushed with highly pure nitrogen gas. All the experiments were performed with a heating rate of 10 °C/min up to 500 °C. The reported values *T*_d5%_ and *T*_d50%_ represent the temperature at which 5% and 50% of the mass is lost, respectively. Concerning the normalization of TGA, a very slight shift (1–2%) in some cases (Figures S41 and S42, SI) took place during the isothermal step at 50 °C which is necessary to stabilize the sample in the oven prior to analysis. The corresponding degradation temperatures reported in this manuscript consider this slight shift (1–2%) while calculating *T*_d5%_ and *T*_d50%_ of the reported polymers.

Differential Scanning Calorimetry (DSC) was performed with a DSC Q20 (TA Instruments). Typically, ~8 mg sample was placed in a sealed pan, flushed with highly pure nitrogen gas and passed through a heat-cool-heat cycle at 10 °C/min in a temperature range of −80 °C to 100 °C. Three heat/cool cycles were done for each sample where the last two cycles were dedicated to analyzing the heat flow of the sample after being cooled in controlled conditions. The *T*_g_ values recorded herein are those obtained from the third cycle.

Fourier-transform infrared spectroscopy (FTIR) was recorded on a Cary 630 FTIR Spectrometer by Agilent (Wilmington, DE, USA).

Liquid chromatography-mass spectrometry (LC-MS) was performed on an Agilent 1290 system, equipped with a PDA UV detector, and a 6545 Q-TOF mass spectrometer (Wilmington, DE, USA). The source was equipped with a JetStream ESI probe operating at atmospheric pressure. The spectrometer was configured according to the following settings: mass range *m/z* 50–1000, gas temperature 325 °C, gas flow 8 L/min, nebulizer 35 psi, sheath gas temperature 350 °C, sheath gas flow 11 L/min, capillary voltage 3500 V, nozzle voltage 2000 V, fragmentor 175 V, skimmer 65 V, octopole RF 750 V. Results were recorded and processed with Mass Hunter B.08.000 software. Elution was performed using a Zorbax Eclipse plus C18 (1.8 µm, 50 × 2.1 mm; Agilent) column heated at 40 °C. The mobile phases were 0.1% formic acid in water (solvent A) and 0.1% formic acid in acetonitrile (solvent B), the flow rate was 0.45 mL/min and followed the gradient: 5% of B during 3 min; 5 to 10% of B from 3rd to 4th min; 10 to 100% of B from 4th to 13th min; 100% of B from 13th to 16th min; 100% to 5% from 16th to 18th min; 5% of B from 18th to 20th min. The sample injection volume was 1 µL and the autosampler was tempered at 10 °C. The UV acquisition was carried out at 250 nm and 320 nm with a reference set at 360 nm.

### 3.1. Synthesis of Monomers

HBO-citro. A biphasic mixture of LGO (50 g, 0.4 mol), citronellol (512 mL, 1.8 mol) and HCl (5 N, 0.6 mol) was stirred at room temperature for 16 h. The resulting mixture was cooled down with an ice bath followed by dropwise addition of a 30% solution of H_2_O_2_ (2 mL) for 2 h. After completion of the addition, the reaction was heated up to 80 °C and stirred for 12 h. The presence of H_2_O_2_ was evaluated with peroxide strips and, if any, the residual H_2_O_2_ was quenched using sodium sulfite. The reaction was extracted with ethyl acetate (two times). Organic layers were washed with brine, dried over anhydrous MgSO_4_, filtered and evaporated to dryness. This step was followed by distillation to remove excess citronellol. The crude product was purified by flash chromatography (gradient 90/10 to 20/80, cyclohexane/ethyl acetate as eluant) to give 62 g of HBO-citro as a pale-yellow oil (58%).


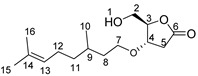


^1^H NMR (δ ppm, CDCl_3_): 5.01 (broad t, *J* = 5.19 Hz, 1H, H_13_), 4.43 (s, 1H, H_3_), 4.11 (d, *J* = 7.0 Hz, 1H, OH), 3.84 (dd, *J* = 3.3 and 12.4 Hz, 1H, H_4_), 3.65 (dd, *J* = 3.3 and 12.4 Hz, 2H, H_2_), 3.39 (m, 2H, H_7_), 2.81 (dd, *J* = 7.0 and 18.1 Hz, 1H, H_5a_), 2.44 (dd, *J* = 3.3 and 18.1 Hz, 1H, H_5s_), 1.90 (m, 2H, H_12_), 1.61 (s, 3H, H_15_), 1.53 (s, 3H, H_16_), 1.27–1.07 (m, 4H, H_11_, H_8_), 0.82 (d, *J* = 6.4 Hz, 3H, H_10_).

^13^C NMR (δ ppm, CDCl_3_): 176.7 (C_6_), 131.2 (C_14_), 124.6 (C_13_), 85.9 (C_3_), 76.1 (C_4_), 67.6 (C_7_), 62.1 (C_2_), 37.1 (C_11_), 36.5 (C_8_), 35.9 (C_5_), 29.3 (C_9_), 25.7 (C_15_), 25.3 (C_12_), 19.4 (C_10_) 17.6 (C_16_).

Triol-citro. An aqueous solution of sodium borohydride (1.14 g in 3 mL of water, 30 mmol) was added dropwise to a solution of HBO-citro (4.5 g, 15 mmol) in THF (60 mL) in a water-ice bath. The reaction mixture was stirred at room temperature for 3 h. The reaction was quenched with acetone and a 20% aqueous solution of citric acid (20 mL). The reaction was extracted twice with ethyl acetate. Organic layers were washed with brine, dried over anhydrous magnesium sulfate, filtered and evaporated to dryness. The crude product was purified by flash chromatography (gradient 90/10 to 20/80, cyclohexane/ethyl acetate as eluant) to give 3.39 g of triol-citro as a colorless oil (82%).


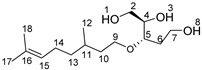


^1^H NMR (δ ppm, DMSO-*d*_6_): 5.07 (t, *J* = 7.2 Hz, 1H, H_15_), 4.55 (s, OH), 4.38 (s, OH), 4.34 (s, OH), 3.50–3.27 (broad, m, 8H, H_9,_ H_7,_ H_5,_ H_4,_ H_2_), 1.93 (broad, m, 2H, H_14_), 1.64 (s, 3H, H_17_), 1.56 (s, 3H, H_18_), 1.50–1.10 (broad, m, 5H, H_13,_ H_11,_ H_10_), 0.84 (d, *J* = 6.2 Hz, 3H, H_12_).

^13^C NMR (δ ppm, DMSO-*d*_6_): 130.8 (C_16_), 125.1 (C_15_), 77.7 (C_5_), 73.5 (C_4_), 67.9 (C_9_), 63.4 (C_2_), 58.2 (C_7_), 37.3 (C_13_), 37.1 (C_10_), 33.9 (C_6_), 29.2 (C_11_), 25.9 (C_17_), 25.4 (C_14_), 19.8 (C_12_), 17.9 (C_18_).

Lactol-citro. A 1.2 M solution of DIBAL-H (27.5 mL of DCM, 33 mmol) was added dropwise to a solution of HBO-citro (4.5 g, 15 mmol) in DCM (68 mL) at −50 °C. The reaction mixture was stirred for 30 min at −50 °C then quenched with a 20% aqueous solution of citric acid (30 mL). The reaction was warmed to room temperature then stirred until the aluminum salt disappeared. Layers were separated and the organic layer was washed with brine, dried over anhydrous magnesium sulfate, filtered and evaporated to dryness. The crude product was purified by flash chromatography (gradient 90/10 to 20/80, cyclohexane/ethyl acetate as eluant) to give 3.63 g of lactol-citro as a colorless oil (89%).

Due to the complexity of the NMR spectra (formation of 5- and 6-membered molecules as well as two diastereomers of each), the interpretation of ^1^H and ^13^C NMR (DMSO-*d*_6_) is provided in Figures S11 and S12 in the Supporting Information.

### 3.2. Polymerizations

Synthesis of P1-P4. A typical two-step melt polycondensation experiment (run 1, Table 2) was performed as follows. Under N_2_ atmosphere, Triol-citro (500 mg, 1.82 mmol) into a 10 mL round bottom flask connected to a vacuum line, equipped with a condensate trap. The flask was cooled down with an ice bath, then malonyl chloride (0.28 mL, 2.82 mmol) was added. The mixture was stirred at room temperature for 19 h. The temperature was then increased gradually up to 50 °C. A high vacuum (10^−3^ bar) was then applied for 3 h. Additional polyesters were prepared with this protocol employing other diacyl chlorides (succinyl chloride, adipoyl chloride and glutaryl chloride).


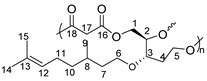


Yield of P1: 95%

^1^H NMR (δ ppm, DMSO-*d*_6_): 5.07 (s, 1H, H_12_), 4.25 (m, 2H, H_1_), 4.13 (m, 1H, H_2_), 3.84–3.48 (m, 6H, H_6_, H_17_, H_5_), 1.94 (m, 2H, H_11_), 1.63 (s, 3H, H_14_), 1.56 (s, 3H, H_15_), 1.50–1.11 (m, 5H, H_10_, H_8_, H_7_), 0.84 (d, *J* = 6.2 Hz, 3H, H_9_).

^13^C NMR (δ ppm, DMSO-*d*_6_):167.1 (C_16_), 129.9 (C_13_), 122.8 (C_12_), 80.8 (C_3_), 79.1 (C_2_), 66.7 (C_6_), 41.1 (C_17_), 36.7 (C_7_), 35.7 (C_10_), 28.4 (C_8_), 25.5 (C_14_), 24.9 (C_11_), 19.4 (C_9_), 17.5 (C_15_).


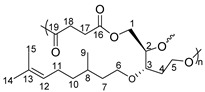


Yield of P2: 84%

^1^H NMR (δ ppm, DMSO-*d*_6_): 5.06 (s, 1H, H_12_), 4.21 (m, 2H, H_1_), 4.07 (m, 1H, H_2_), 3.81–3.41 (m, 5H, H_3_, H_6_, H_5_), 2.89 (s, 2H, H_18_), 2.41 (s, 2H, H_17_), 1.67 (m, 2H, H_11_), 1.52 (s, 6H, H_15_, H_14_), 1.26–1.09 (m, 5H, H_10_, H_8_, H_7_), 0.83 (d, *J* = 6.2 Hz, 3H, H_9_).

^13^C NMR (δ ppm, DMSO-*d*_6_): 173.7 (C_19_), 173.2 (C_16_), 78.7 (C_3_), 73.5 (C_2_), 68.2 (C_6_), 66.9 (C_5_), 62.2 (C_1_), 45.6 (C_4_), 36.6 (C_7_), 36.4 (C_10_), 32.2 (C_8_), 28.9 (C_17_), 22.1 (C_11_), 19.4 (C_9_).


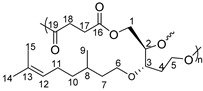


Yield of P3: 88%

^1^H NMR (δ ppm, DMSO-*d*_6_): 5.09 (s, 1H, H_12_), 4.29 (m, 2H, H_1_), 4.06 (m, 1H, H_2_), 3.57–3.39 (m, 4H, H_6_, H_5_), 2.29–2.20 (m, 6H, H_19,_ H_18,_ H_17_), 1.67 (m, 2H, H_11_), 1.52 (s, 3H, H_14_), 1.48 (s, 3H, H_15_), 1.24–1.08 (m, 5H, H_10_, H_8_, H_7_), 0.81 (d, *J* = 6.2 Hz, 3H, H_9_).

^13^C NMR (δ ppm, DMSO-*d*_6_): 174.4 (C_20_), 45.6 (C_4_), 36.6 (C_7_), 36.4 (C_10_), 33.5 (C_19_), 32.17 (C_8_), 28.9 (C_17_), 24.1 (C_14_), 22.0 (C_11_), 19.4 (C_9_).


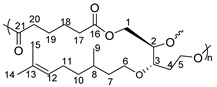


Yield of P4: 87%

^1^H NMR (δ ppm, DMSO-*d*_6_): 5.12 (s, 1H, H_12_), 4.29 (m, 2H, H_1_), 4.06 (m, 1H, H_2_), 3.54–3.39 (m, 4H, H_6_, H_5_), 2.70 (t, *J* = 6.6 Hz, 2H_,_ H_20_), 2.23 (t, *J* = 6.6 Hz, 2H, H_17_), 1.69 (m, 2H, H_11_), 1.53 (s, 6H, H_15_, H_14_), 1.28–1.10 (m, 5H, H_10_, H_8_, H_7_), 0.82 (d, *J* = 6.2 Hz, 3H, H_9_).

^13^C NMR (δ ppm, DMSO-*d*_6_): 174.1 (C_16_), 45.5 (C_4_), 36.3 (C_10_), 33.4 (C_17_), 29.4 (C_8_), 22.8 (C_19_), 19.9 (C_9_), 15.7 (C_15_).

### 3.3. Enzymatic Degradation

Prior to its use, the enzyme activity was assessed using the *p*-nitrophenyl butyrate colorimetric assay. Absorbance was monitored at 400 nm and the results were compared with a *p*-nitrophenol calibration curve. P1-P4 were ground using a spatula to yield powdered polyester samples. 50 mg of the ground samples were placed in 4 sealed vials containing phosphate buffer (3 mL, 0.05 M) and 10 mg/mL of Lipopan^®^ 50 BG (50 KLU/g). The enzyme concentration was adapted from Alejandra et al. [38]. The mixtures were then incubated at the pH and temperature optima for the enzyme (37 °C, pH 7) and stirred gently at 50 rpm for predetermined time intervals. The hydrolyzed polymers were washed three times with 10 mL of water followed by centrifugation for 10 min at 10 °C and 4750 rpm. The resulting products were freeze-dried to remove all traces of water before being subjected to analysis. All isolated powders were analyzed by ^1^H NMR, SEC, DSC and FTIR. Controls were realized for each polymer in phosphate buffer solution without enzyme and showed no degradation.

## 4. Conclusions

LGO was used as a starting material to produce new renewable citronellol-containing molecules. The acid-catalyzed oxa-Michael addition of citronellol to LGO, followed by the solvent-free H_2_O_2_-mediated Baeyer-Villiger oxidation, led to a novel versatile functional lactone, HBO-citro. The reduction of HBO-citro was investigated using two types of reducing agents: i) NaBH_4_ which led selectively to the synthesis of citronellol-containing tris-hydroxy monomer, Triol-citro, and ii) DIBAL-H where a mixture of 5- and 6-membered cyclic forms of Lactol-citro was obtained. Other reducing agents equivalent to DIBAL-H (e.g., LiAlH_4_/BH_3_.THF, LiBH_4_, Red.Al, LiEt_3_BH) and to NaBH_4_ (e.g., NBH_3_CN), could have been possibly tested. Each of them has its pros and cons. However, as the straightforward synthesis of Triol-citro was achieved selectively using NaBH_4_—which is safer and less toxic than DIBAL-H—NaBH_4_ can be adopted for the preparation of Triol-citro from HBO-citro.

The solvent-free polycondensation of Triol-citro with aliphatic diacyl chlorides was then sought. Branched/crosslinked functional polyester structures were formed. The obtained polymers showed promising properties including good thermal stabilities with *T*_d5%_ up to 170 °C and low *T*_g_ ranging from −20 °C to −42 °C depending on the (co-)monomer used.

In an attempt to assess the biodegradation of the polyesters, P1-P4 were then subjected to the enzymatic degradation in the presence of Lipopan^®^ 50 BG. An impact of polymer structure as well as of the co-monomer chain length on the enzyme accessibility and degradation profile was observed. From a general point of view, SEC, DSC, ^1^H NMR and FTIR analyses showed that the ester bonds of P2-P4 have a high tendency to be hydrolyzed when a lipase such as Lipopan^®^ 50 BG is present.

The synthesis and characterization of other fully renewable citronellol-containing polymers with different structures, higher solubility and controlled degree of branching, as well as the effect of citronellol photocrosslinking on the polymer properties, are currently under investigation and will be reported shortly in a forthcoming full paper.

## Figures and Tables

**Table 1:** Reduction of HBO-citro.

Run ^1^	Reducing Agent (eq) ^2^	Solvent	Time (h)	Product	Yield (%) ^3^
1^4^	NaBH_4_ (2)	THF	2	Triol-citro	54
2	NaBH_4_ (2)	THF	2	Triol-citro	79
3	NaBH_4_ (2)	THF	3	Triol-citro	82
4	NaBH_4_ (2)	2-MeTHF	3	Triol-citro	35
5	NaBH_4_ (2)	2-MeTHF	20	Triol-citro	68
6	NaBH_4_ (2)	EtOH	3	Triol-citro	56
7	NaBH_4_ (2)	EtOH	26	Triol-citro	63
8 ^4^	DIBAL-H (2)	DCM	0.5	Mixture	44
9	DIBAL-H (2.2)	DCM	0.5	Mixture	89
10	DIBAL-H (2.2)	2-MeTHF	0.5	Mixture	41
11	DIBAL-H (2.2)	2-MeTHF	3	Mixture	22

^1^ [HBO-citro] = 1.2 M, T = room temperature (20 °C) (runs 1–7) or −50 °C (runs 8–11). ^2^ Number of equivalents of reducing agent based on HBO-citro. ^3^ Yield of the purified product. ^4^ Reactions were performed under air.

**Table 2:** Polycondensation of Triol-citro with acyl chloride derivatives.

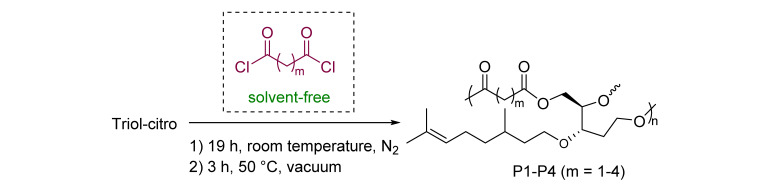						
Run	Polymer	*M*_n_ (kDa) ^1^	*Đ* ^1^	*T*_g_ (°C) ^2^	*T_d_*_5%_ (°C) ^3^	*T_d_*_50%_ (°C) ^4^
1	P1	3.4	1.6	−20	142	320
2	P2	2.4	1.6	−22	152	358
3	P3	1.8	1.3	−42	165	388
4	P4	1.6	1.1	−20	170	386

^1^ Determined in DMF (10 mM LiBr) at 50 °C, only showed the soluble parts of the polymers. ^2^ Glass transition determined by DSC. ^3^ TGA degradation temperature at which 5% (*T*_d5%_) mass loss was observed under nitrogen. ^4^ TGA degradation temperature at which 50% (*T*_d50%_) mass loss was observed under nitrogen.

**Table 3:** Enzymatic degradation of P1-P4 using Lipopan^®^ 50 BG.

Run ^1^	Polymer	*M*_n_ (kDa) ^2^	*T*_g_ (°C) ^3^	^1^H NMR ^4^	FTIR ^5^
1	P1	3.4	−30	✗	✗
2	P2	NS ^6^	-	-	✓
3	P3	0.8	−22	✓	✓
4	P4	NS ^6^	−14	✓	✓

^1^ Runs 1–4 are listed following the same order of Table 2. ^2^ Determined in DMF (10 mM LiBr) at 50 °C. ^3^ Glass transition determined by DSC, temperature ramp 10 °C/min. ^4 1^H NMR of the hydrolyzed product in DMSO-*d*_6_, ✓: presence of hydroxy signal(s), ✗: absence of hydroxy signal. ^5^ Infra-red of the hydrolyzed product, ✓: presence of hydroxy band, ✗: absence of hydroxy band. ^6^ NS: no signal was observed within the calibrated retention time interval.

## Data Availability

Not applicable.

## Supplementary Materials

The following are available online, NMR (Figures S1–S24), LC-MS (Figures S25 and S26), SEC (Figures S27–S34), FT-IR (Figures S35 and S36), DSC (Figures S37–S40) and TGA (Figures S41–S44).

## Sample Availability

Samples of the compounds are not available from the authors.
