# Supplementary material for: Unprecedented Biodegradable Cellulose-Derived Polyesters with Pendant Citronellol Moieties: From Monomer Synthesis to Enzymatic Degradation
Source: Molecules. 2021 Dec 18;26(24):7672. doi: 10.3390/molecules26247672 (PMC8707784; doi:10.3390/molecules26247672)
Supplement: Supplementary file 1 [file molecules-26-07672-s001.zip › molecules-1490869-supplementary.pdf]

Supporting Information

Unprecedented biodegradable cellulose-derived polyesters having pendant citronellol moieties: from monomer synthesis to enzymatic degradation

Aihemaiti Kayishaer, Sami Fadlallah,* Louis M. M. Mouterde,* Aurélien A. M. Peru, Yasmine Werghi, Quentin Carboué, Michel Lopez, Florent Allais*

URD Agro-Biotechnologies Industrielles (ABI), CEBB, AgroParisTech, 51110 Pomacle.

* sami.fadlallah@agroparistech.fr, louis.mouterde@agroparistech.fr, florent.allais@agroparistech.fr

Table of contents

NMR 2
LC-MS 16
SEC 17
FT-IR 21
DSC 22
TGA 24

I. NMR

a. HBO-citro

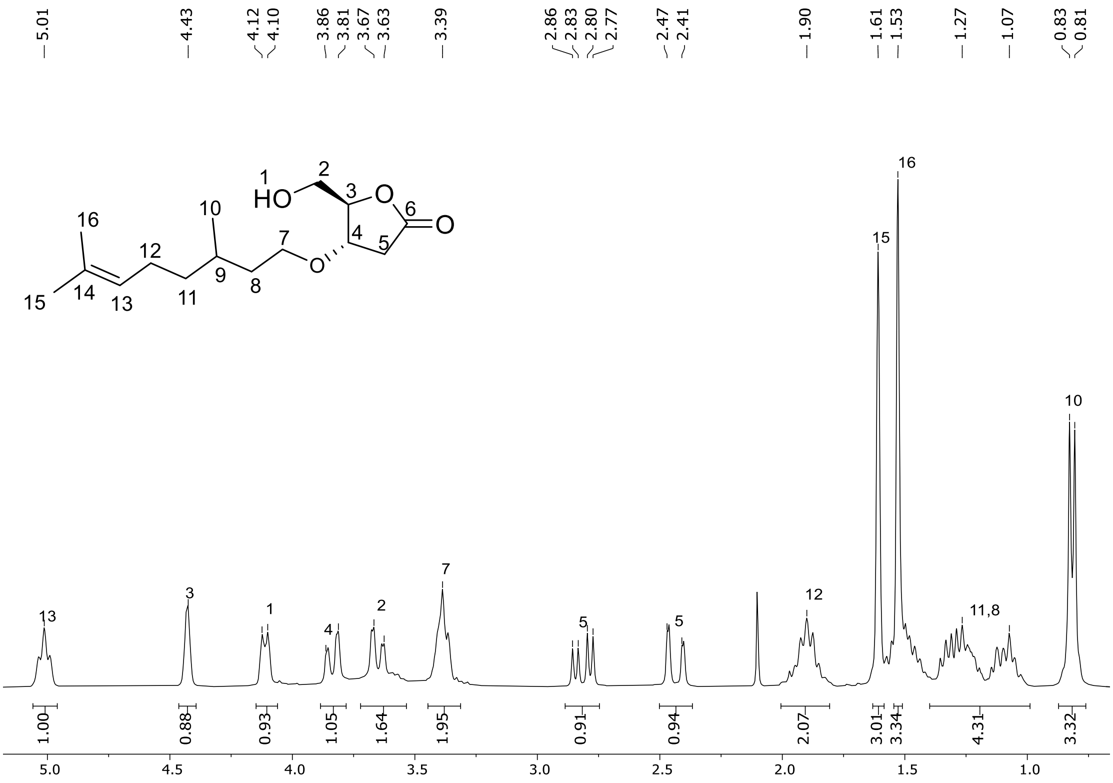

Figure S1. ^{1}H NMR ($CDCl_3$) spectrum of HBO-citro

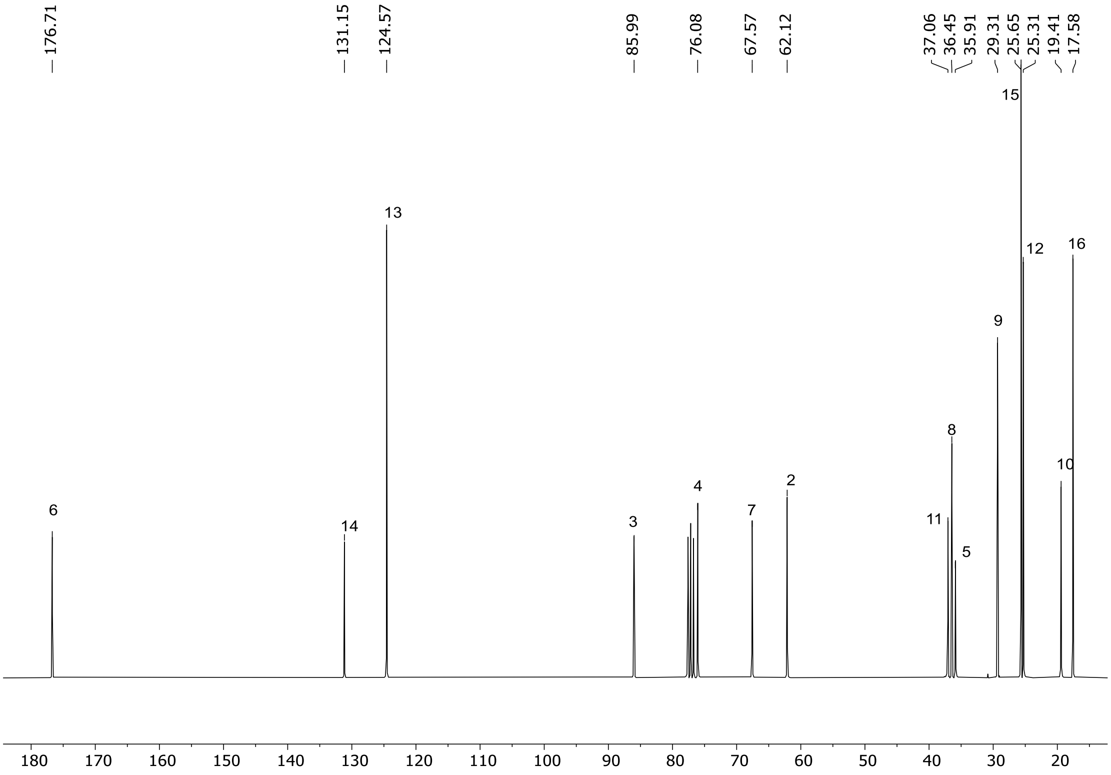

Figure S2. ^{13}C NMR ($CDCl_3$) spectrum of HBO-citro

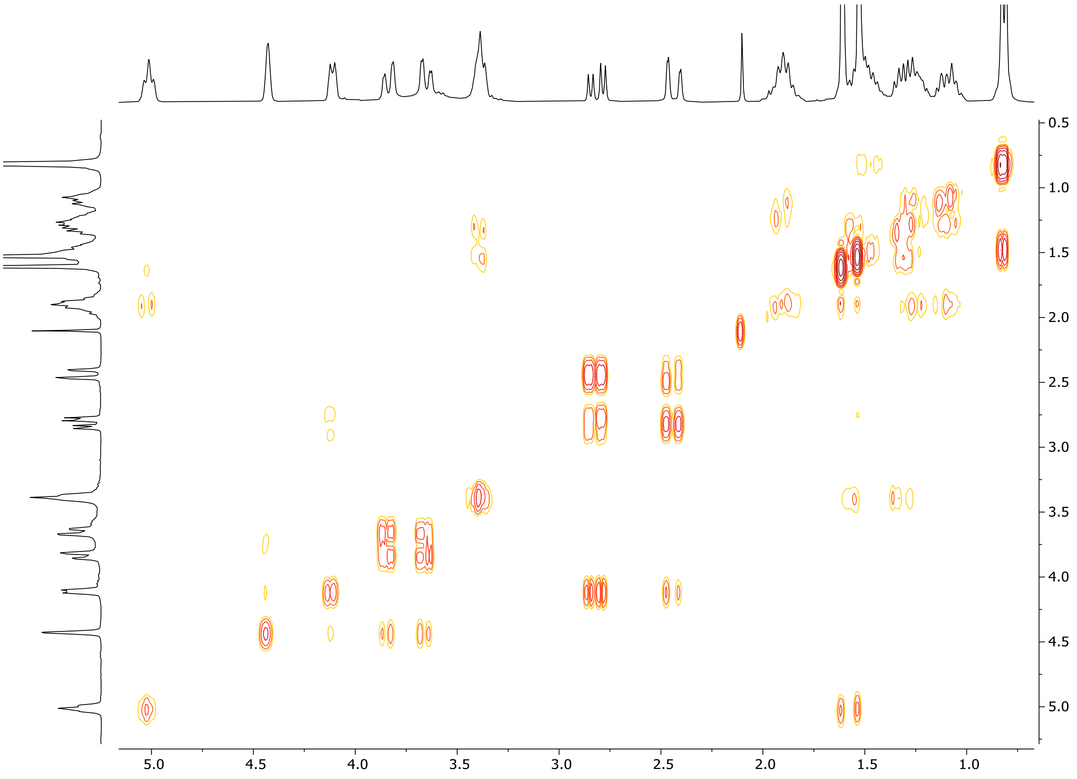

Figure S3. ^{1}H-^{1}H COSY ($CDCl_3$) spectrum of HBO-citro

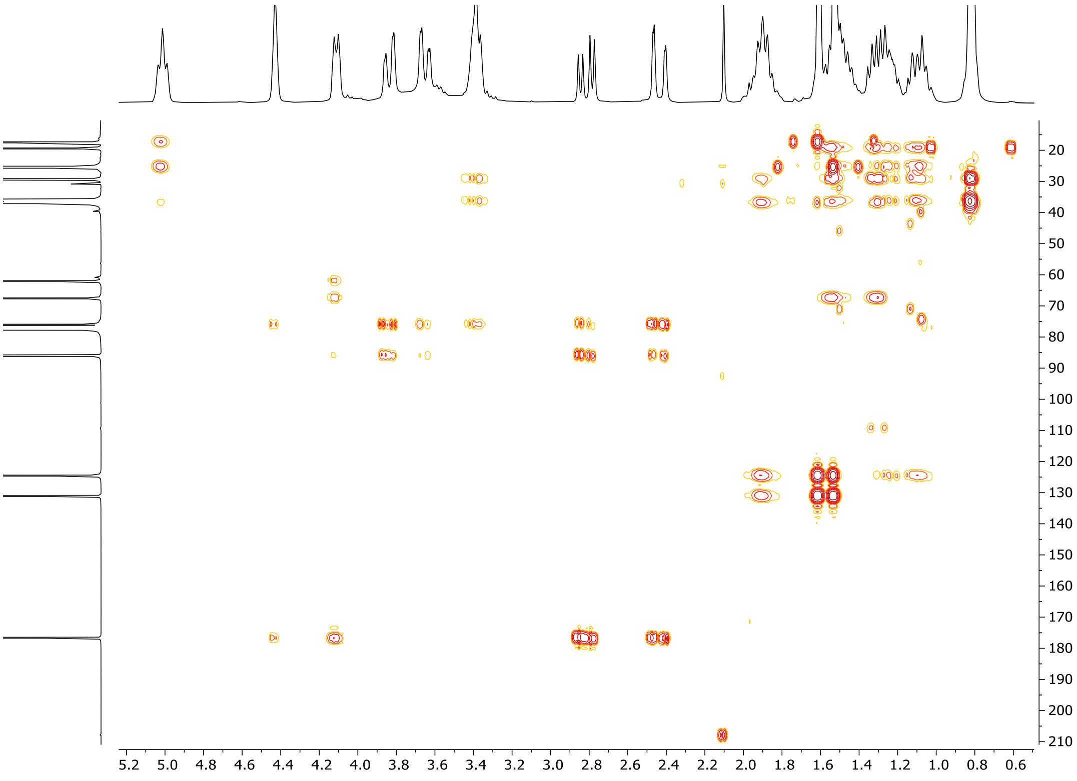

Figure S4. ^{1}H-^{13}C HMBC ($CDCl_3$) spectrum of HBO-citro

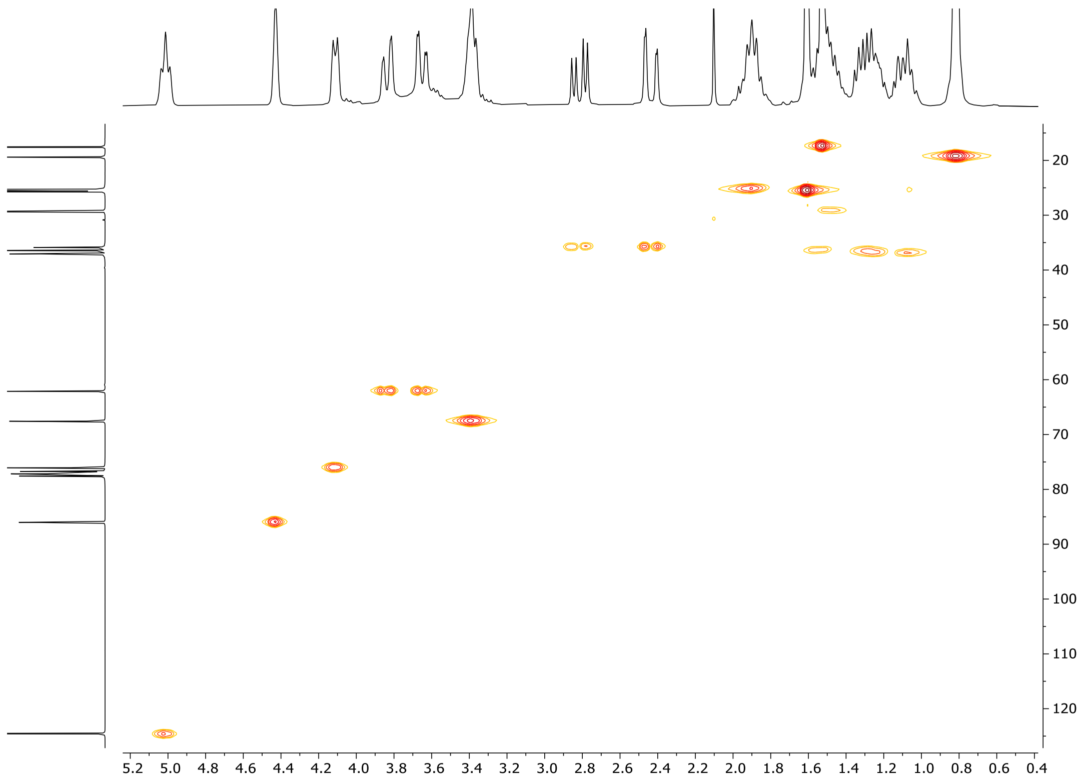

Figure S5. ^{1}H-^{13}C HSQC ($CDCl_3$) spectrum of HBO-citro

b. Triol-citro

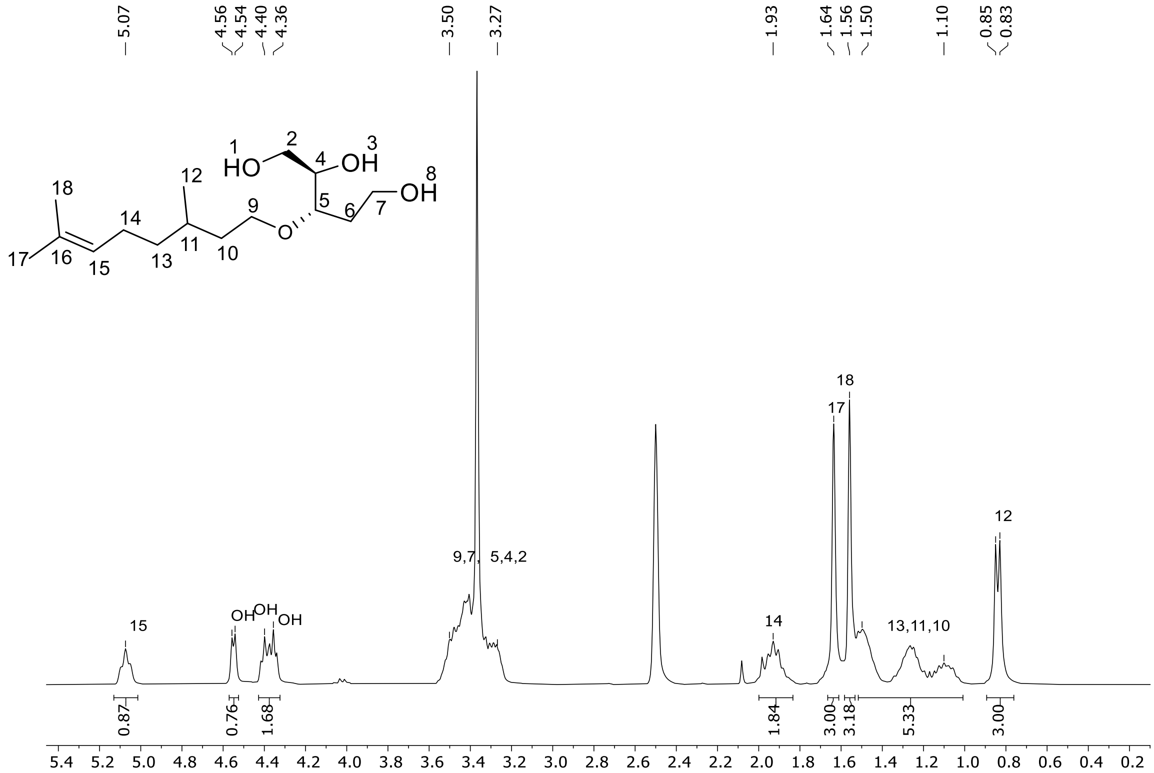

Figure S6. ^{1}H NMR (DMSO-d_6) spectrum of Triol-citro

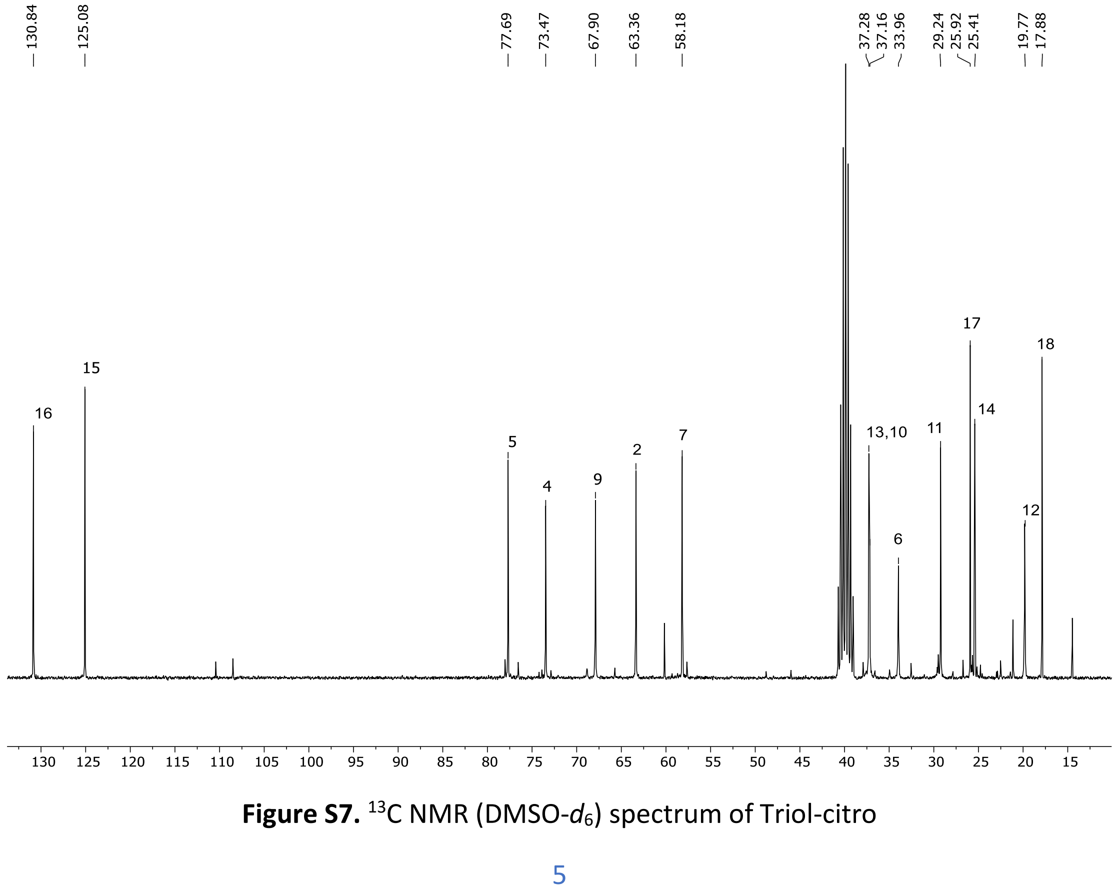

Figure S7. ^{13}C NMR (DMSO-d_6) spectrum of Triol-citro

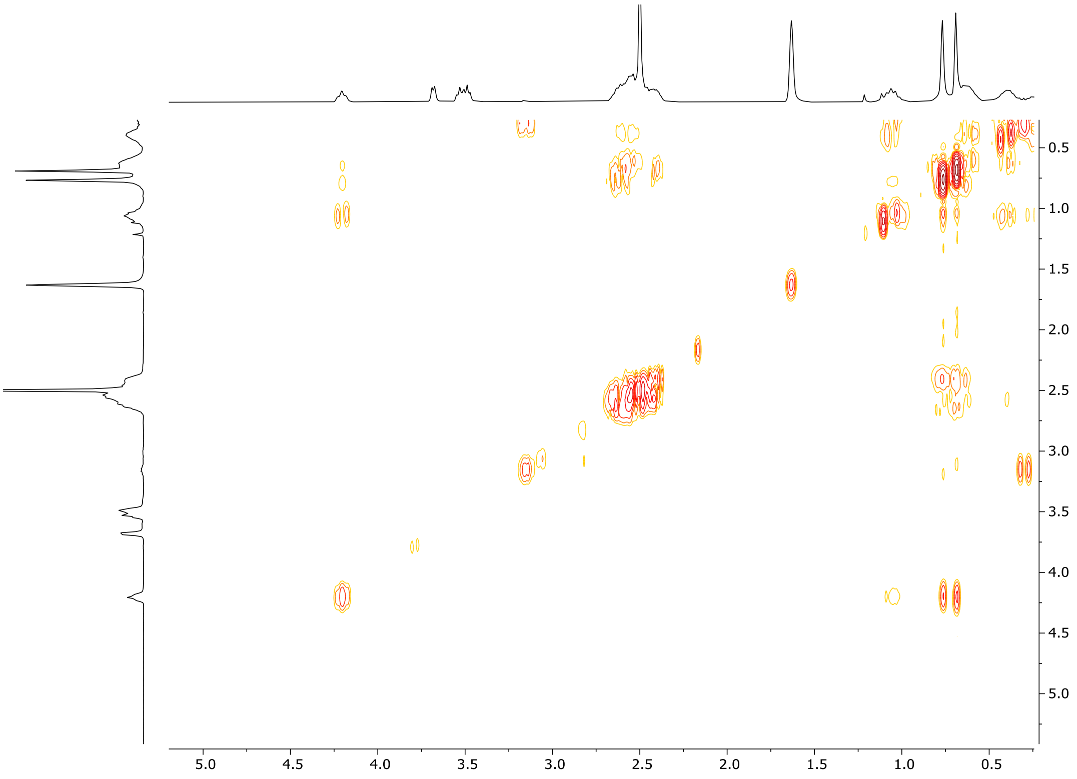

Figure S8. ^{1}H-^{1}H COSY (DMSO-d_6) spectrum of Triol-citro

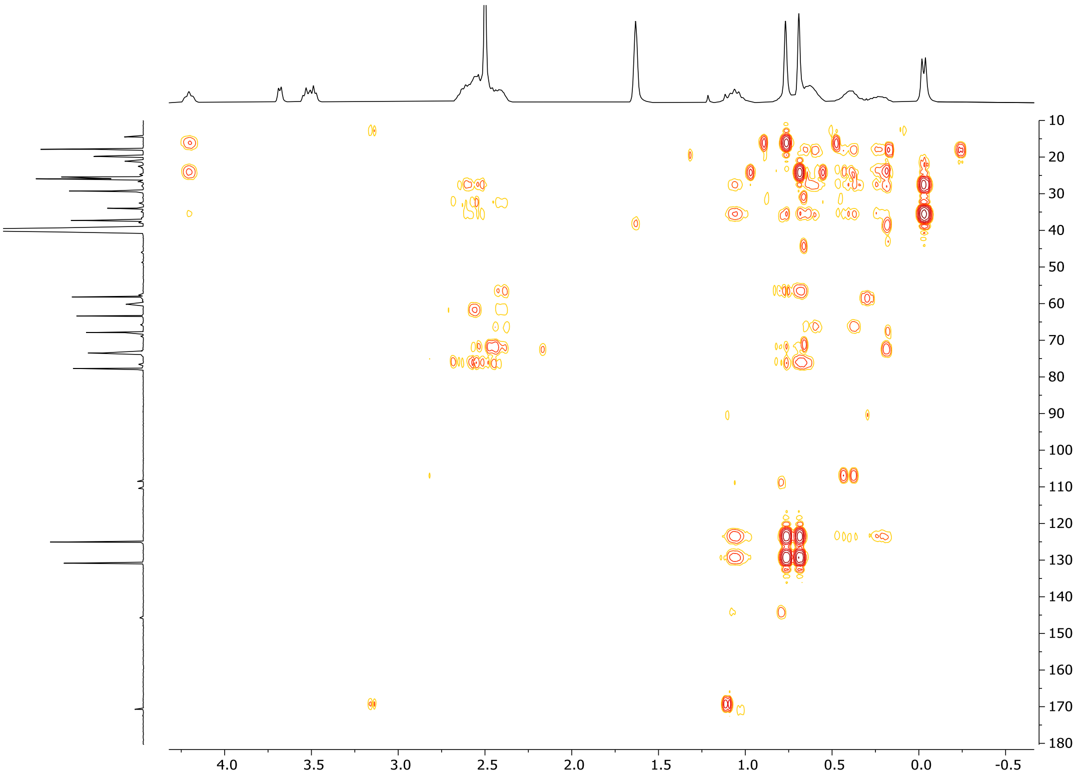

Figure S9. ^{1}H-^{13}C HMBC (DMSO-d_6) spectrum of Triol-citro

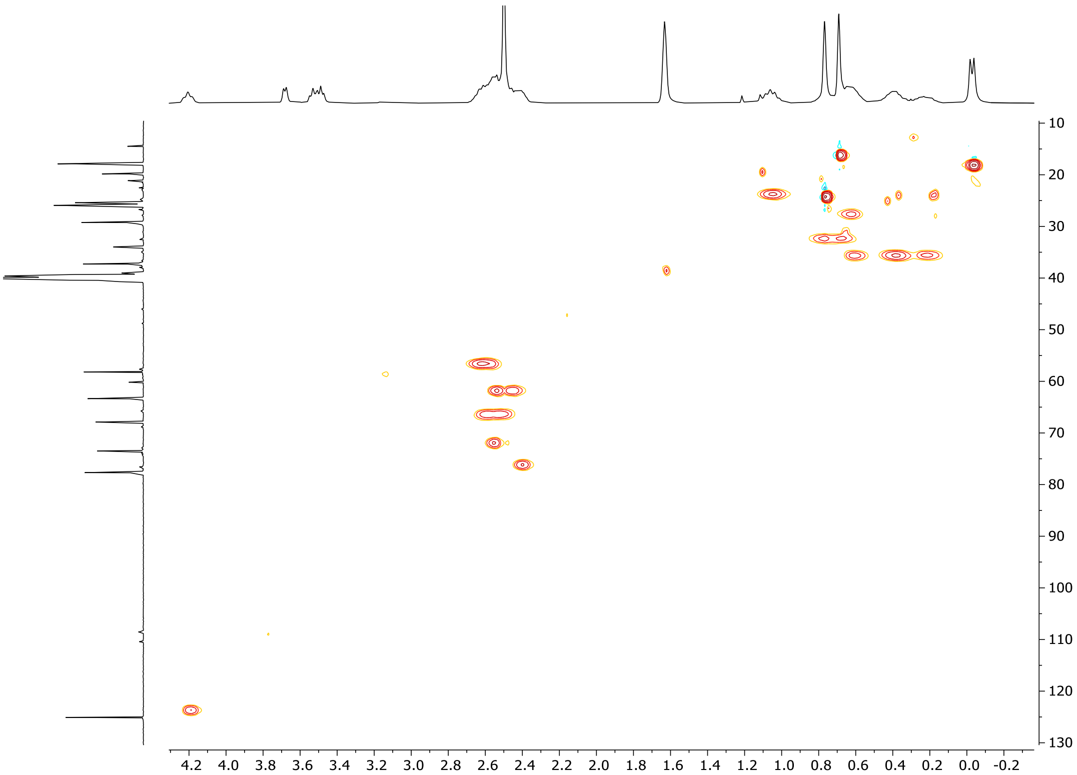

Figure S10. ^{1}H-^{13}C HSQC (DMSO-d_6) spectrum of Triol-citro

c. Lactol-citro mixture

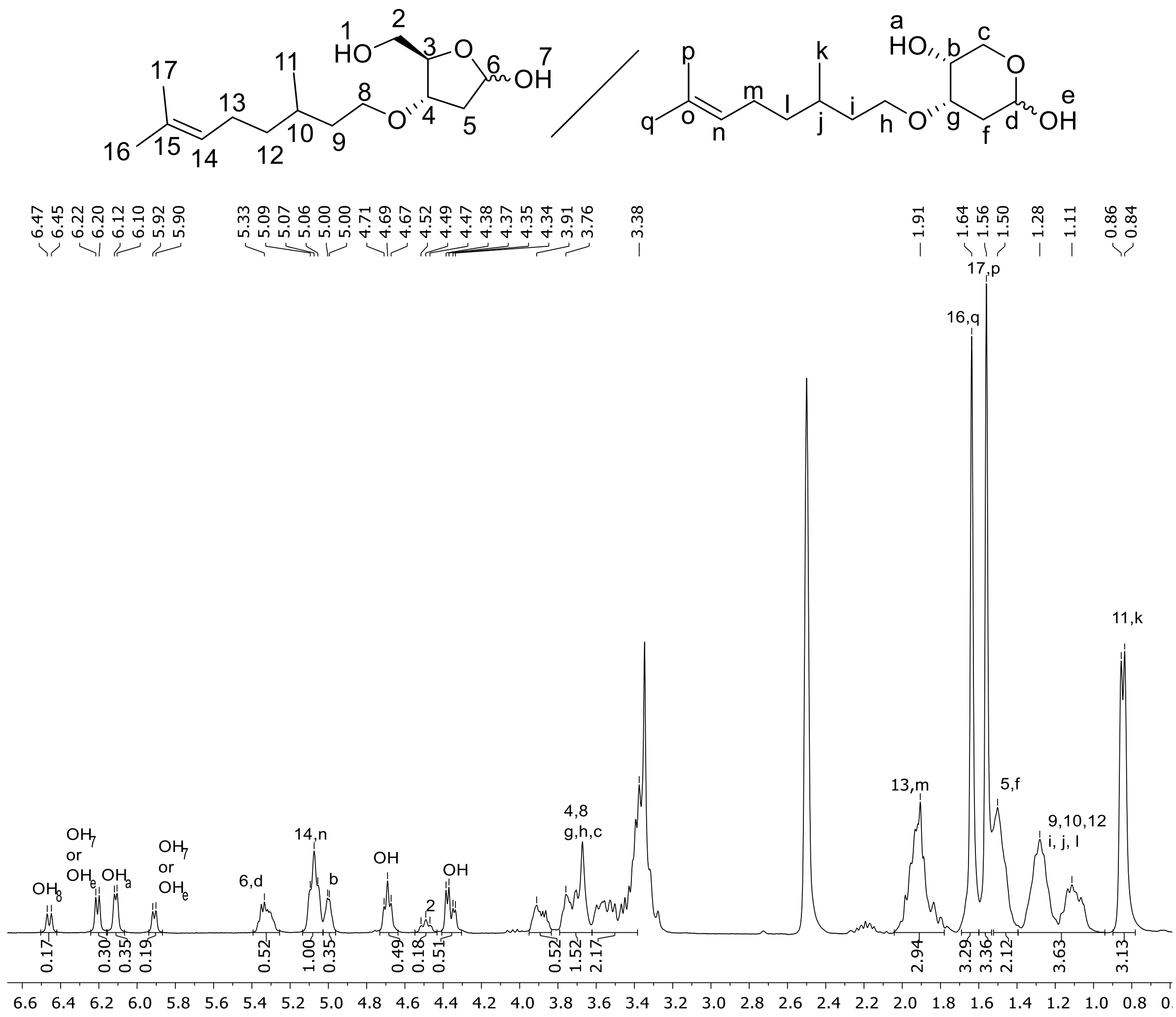

Figure S11. ¹H NMR (DMSO-d_6) spectrum of Lactol-citro

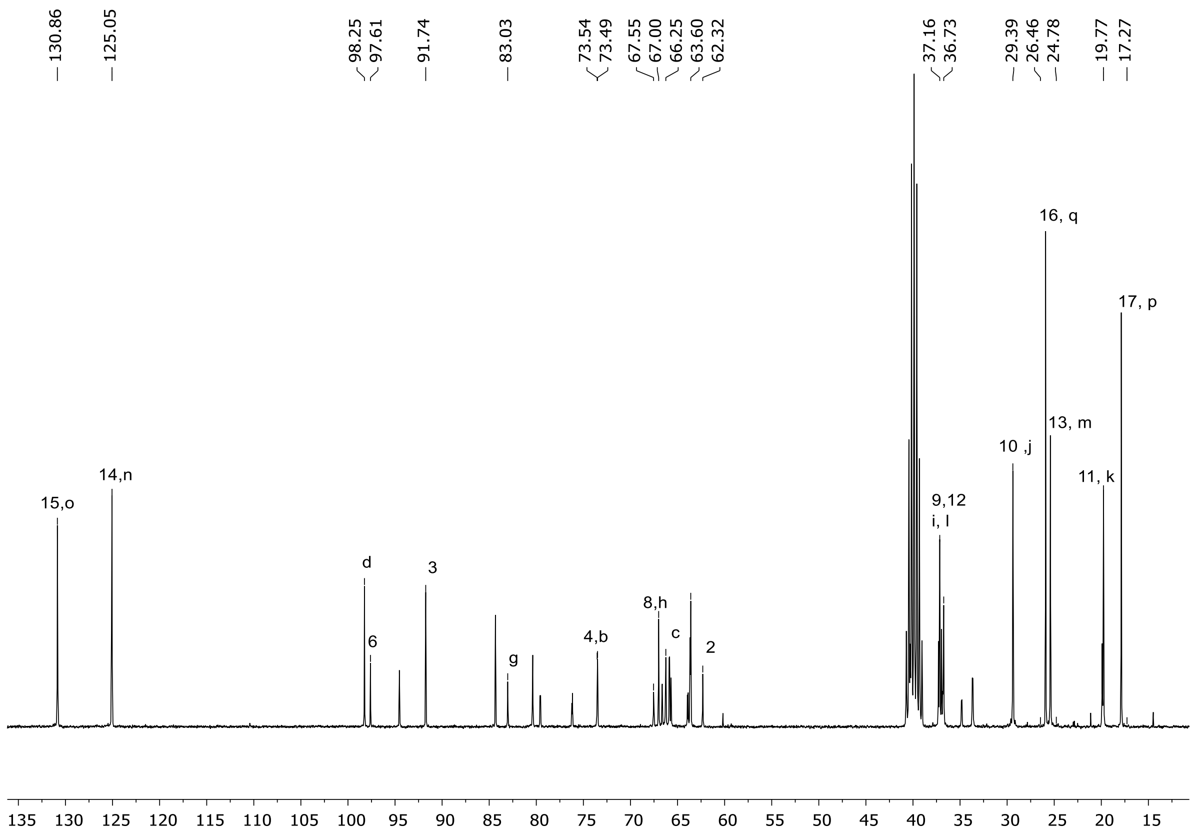

Figure S12. ^{13}C NMR (DMSO-d_6) spectrum of Lactol-citro

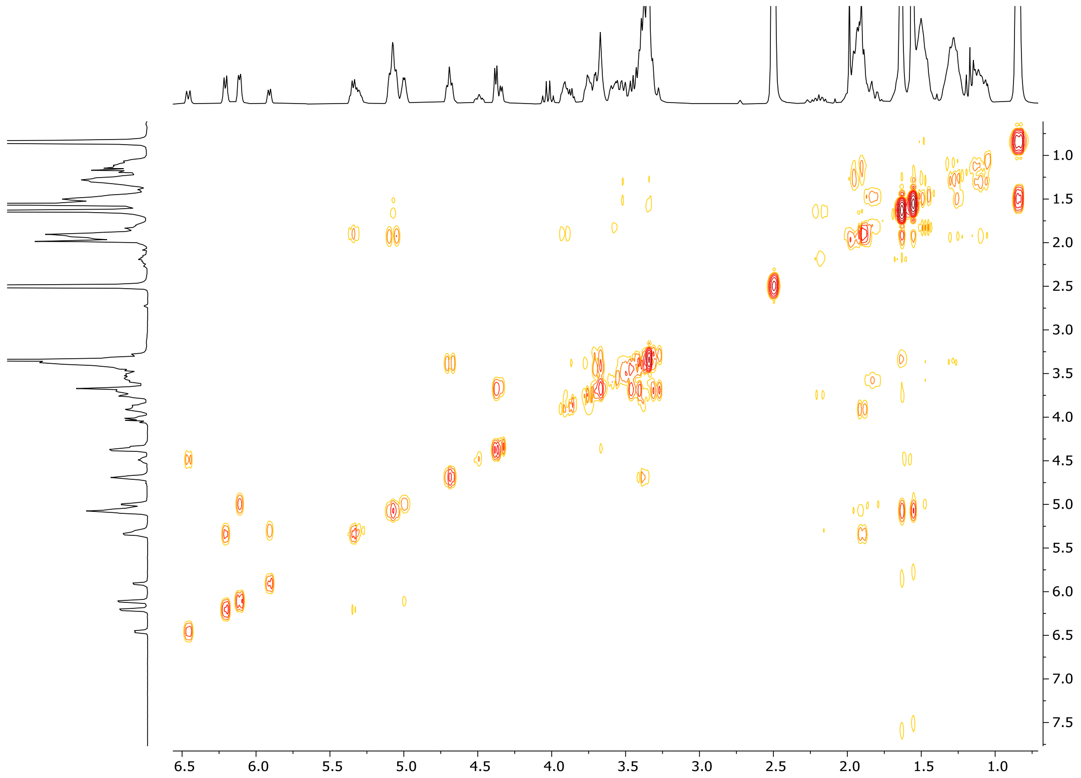

Figure S13. ^{1}H-^{1}H COSY (DMSO-d_6) spectrum of Lactol-citro

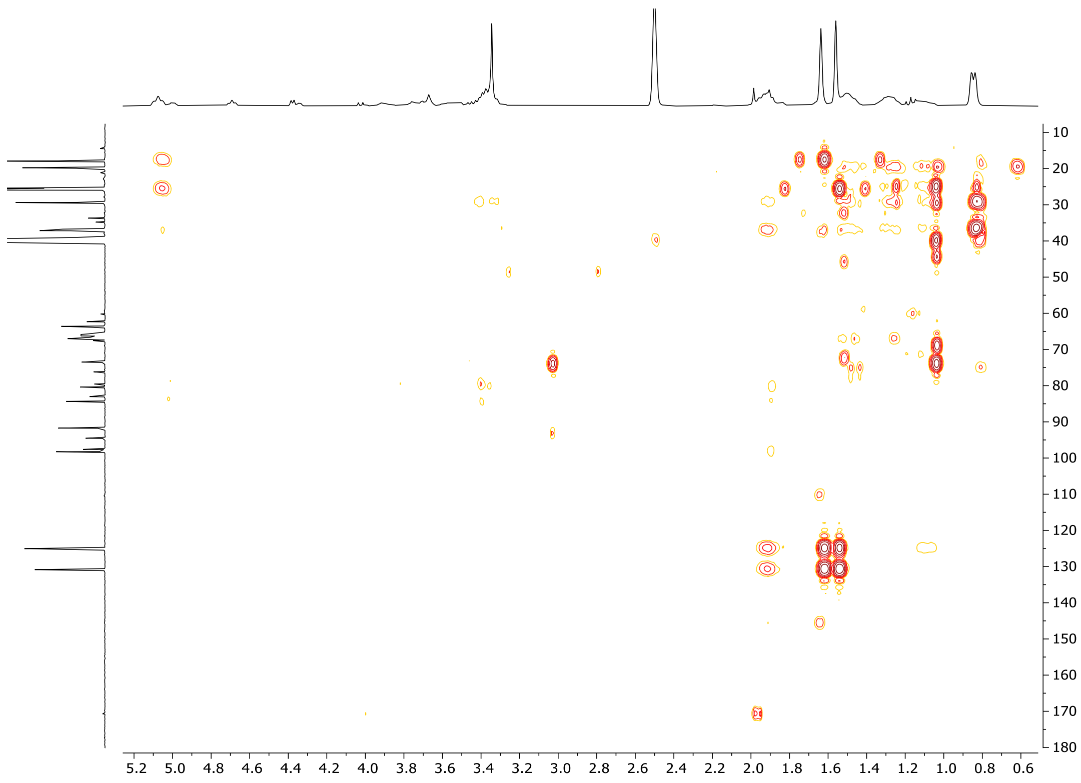

Figure S14. ^{1}H-^{13}C HMBC (DMSO-d_6) spectrum of Lactol-citro

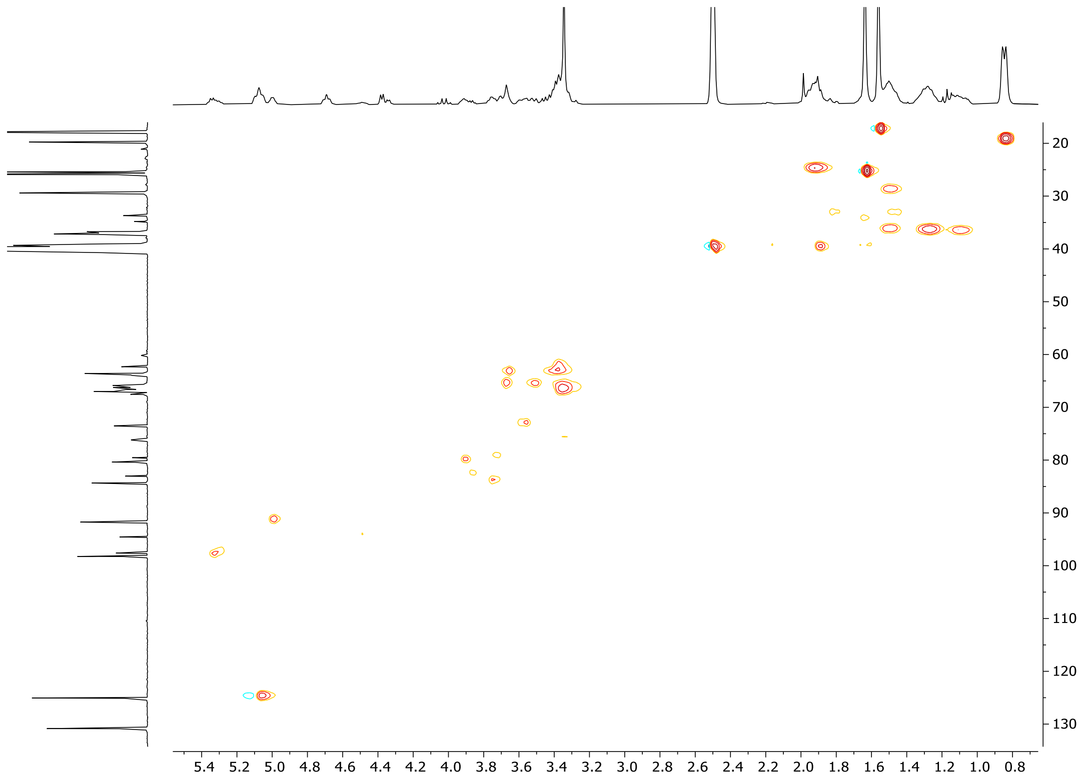

Figure S15. ^{1}H-^{13}C HSQC (DMSO-d_6) spectrum of Lactol-citro

d. P1

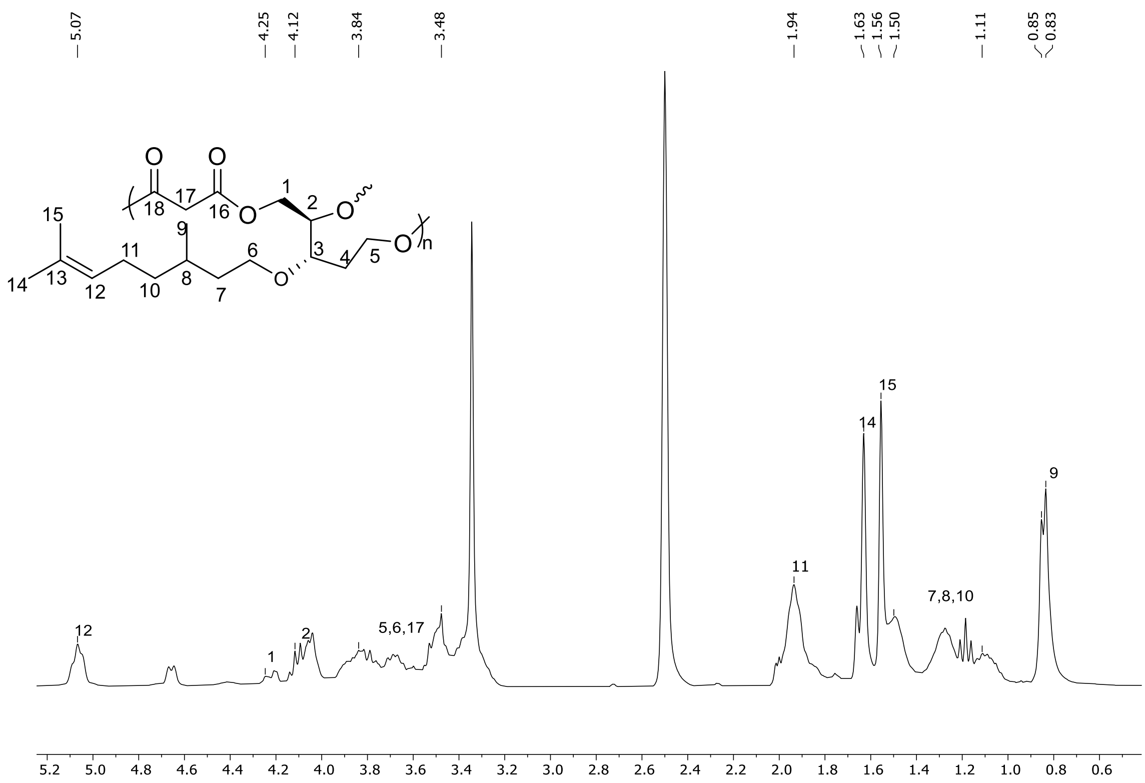

Figure S16. Typical ^{1}H NMR (DMSO-d_6) spectrum of P1

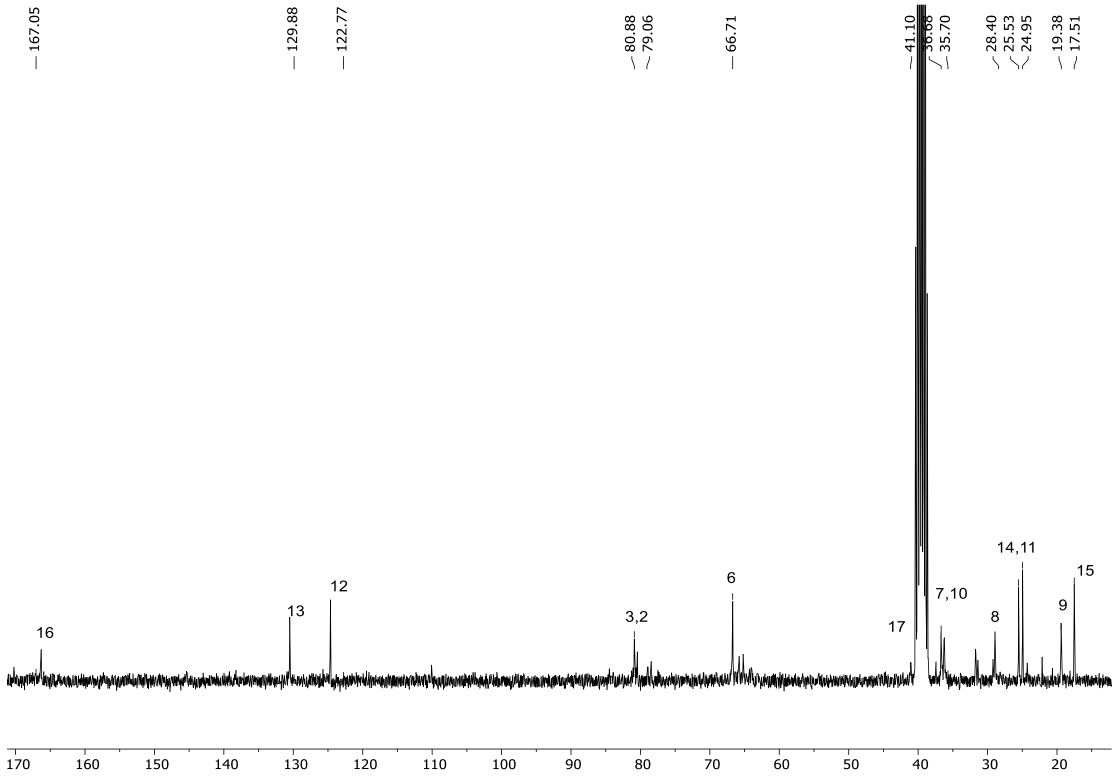

Figure S17. Typical ^{13}C NMR (DMSO-d_6) spectrum of P1

e. P2

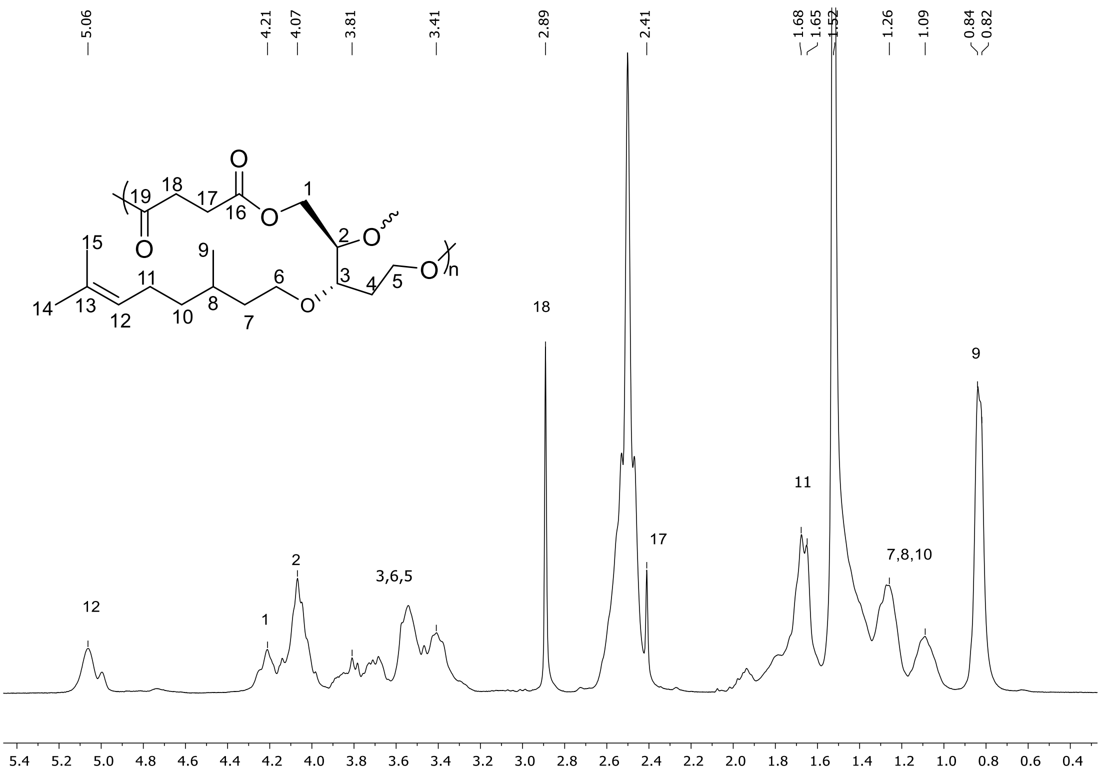

Figure S18. Typical ^{1}H NMR (DMSO-d_6) spectrum of P2

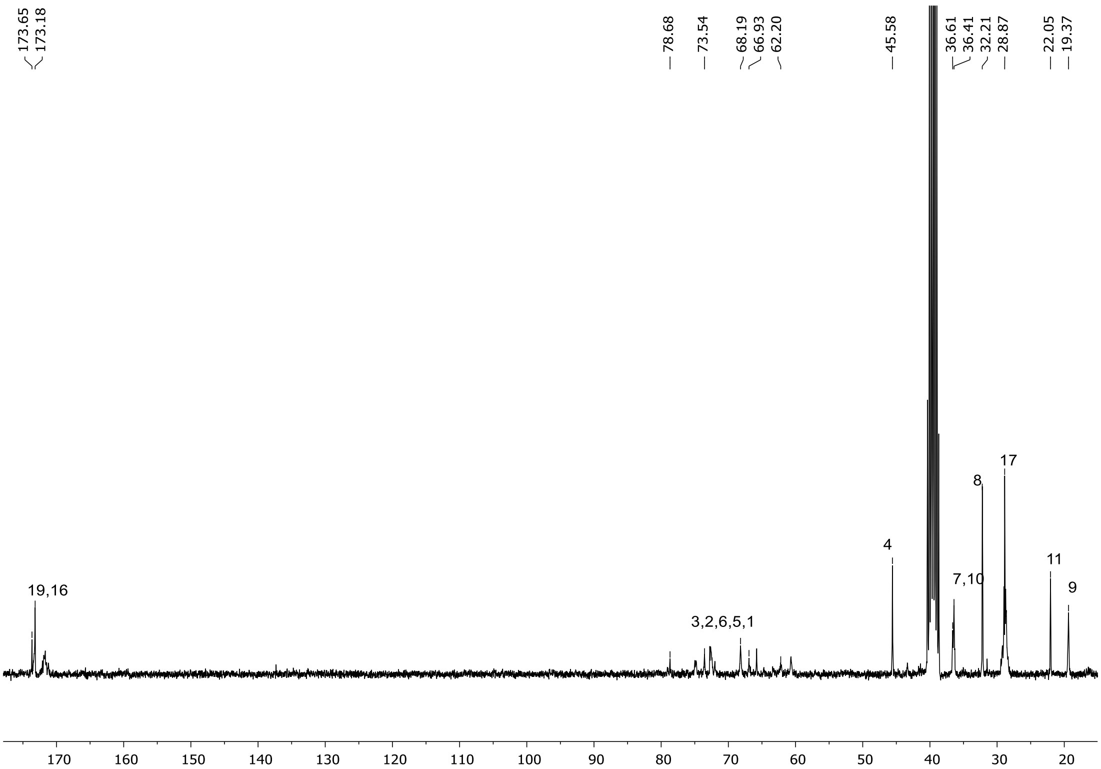

Figure S19. Typical ^{13}C NMR (DMSO-d_6) spectrum of P2

f. P3

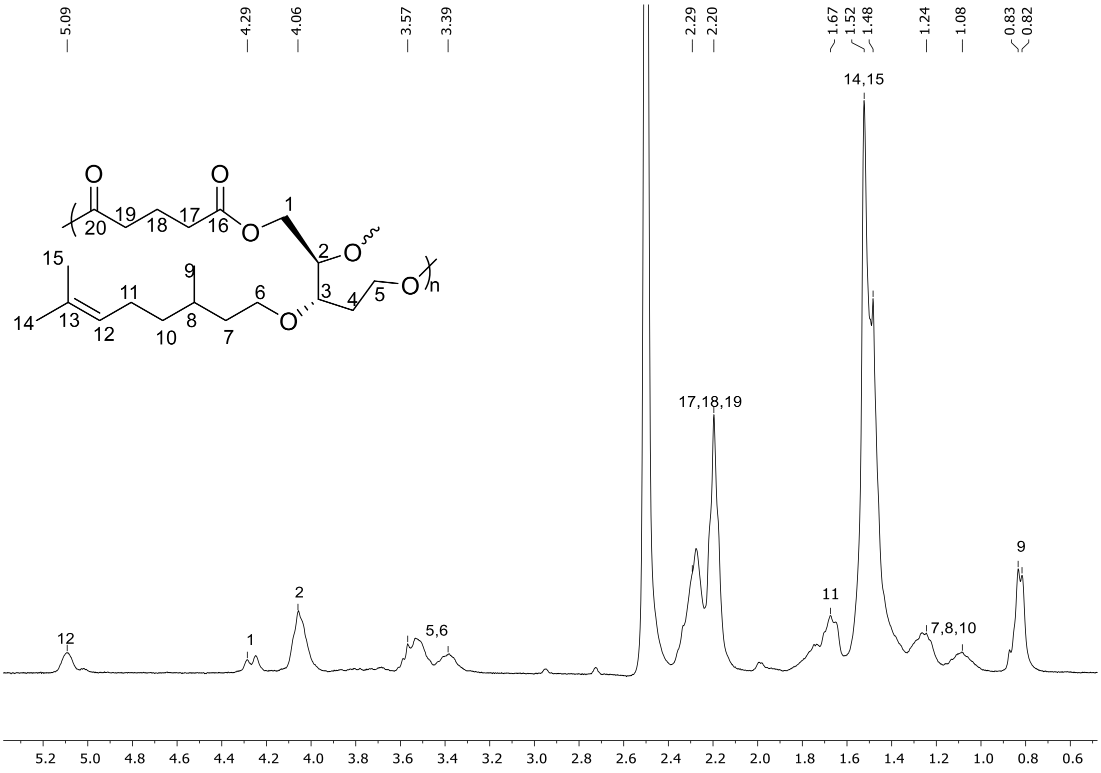

Figure S20. Typical ^{1}H NMR (DMSO-d_6) spectrum of P3

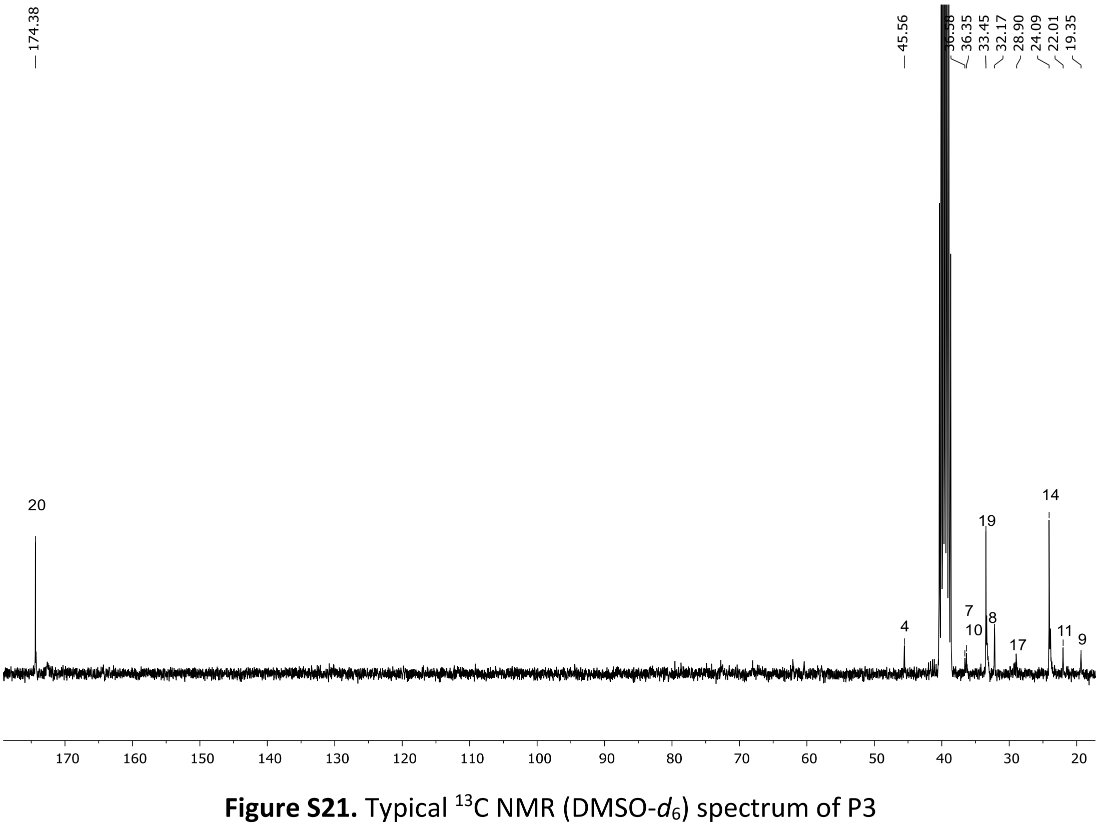

Figure S21. Typical ^{13}C NMR (DMSO-d_6) spectrum of P3

g. P4

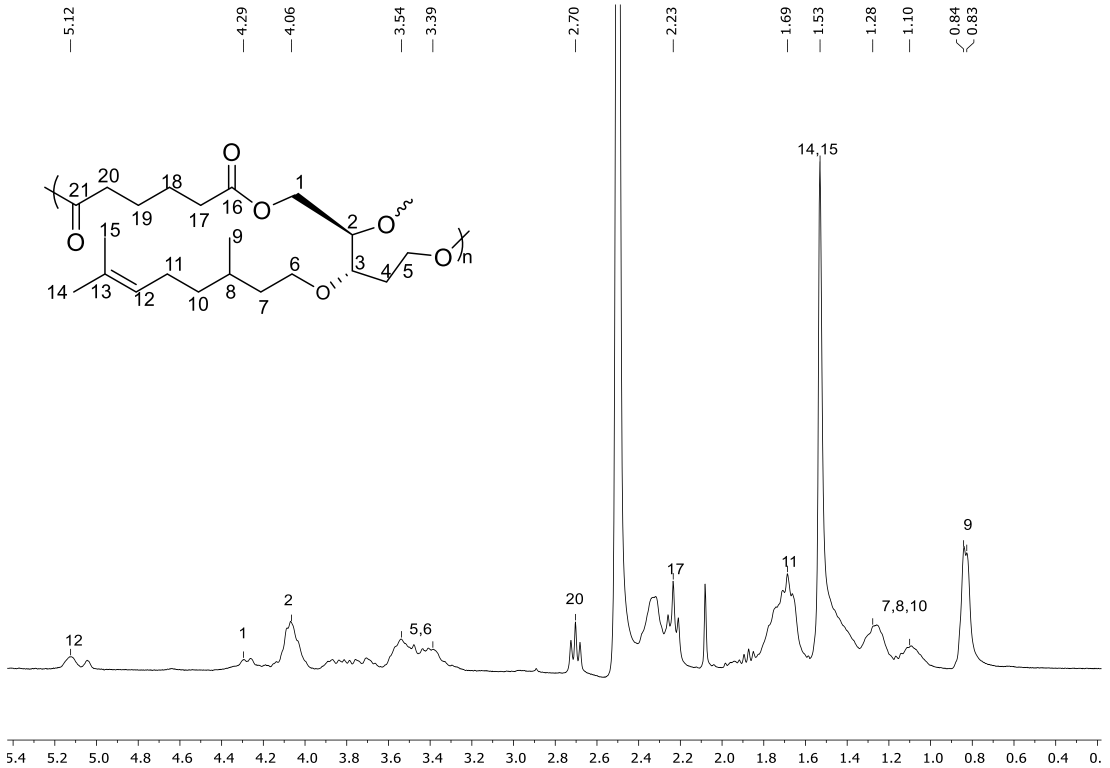

Figure S22. Typical ^{1}H NMR (DMSO-d_6) spectrum of P4

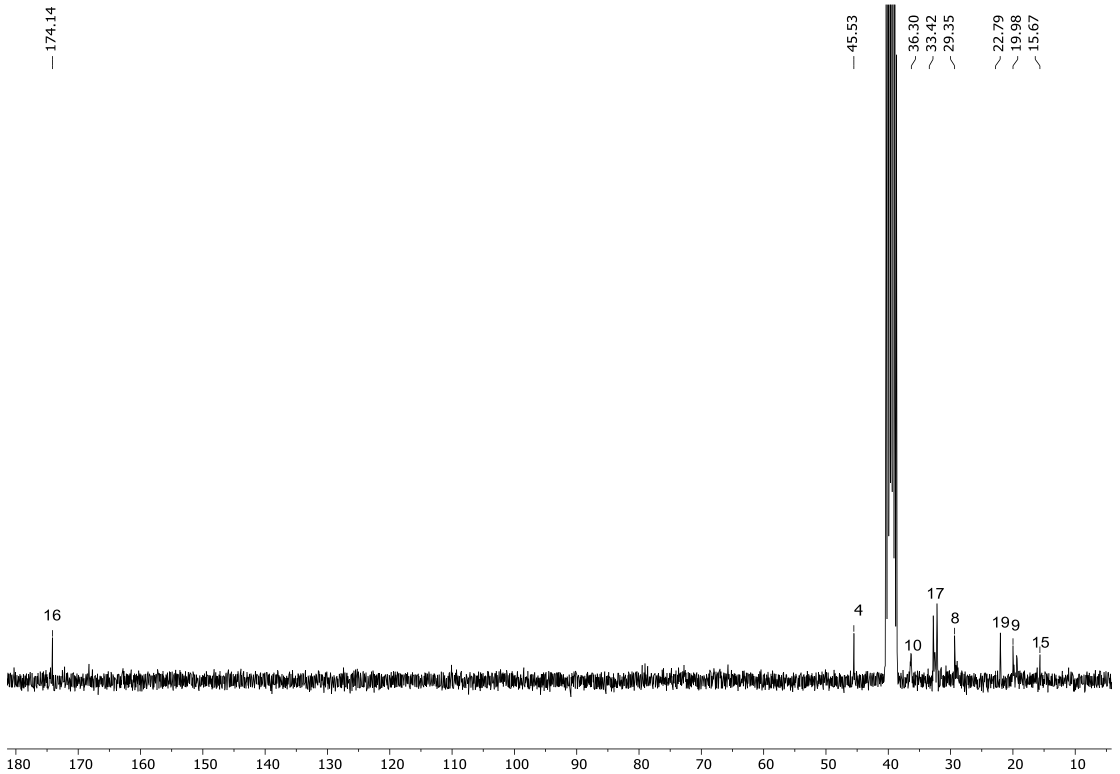

Figure S23. Typical ^{13}C NMR (DMSO-d_6) spectrum of P4

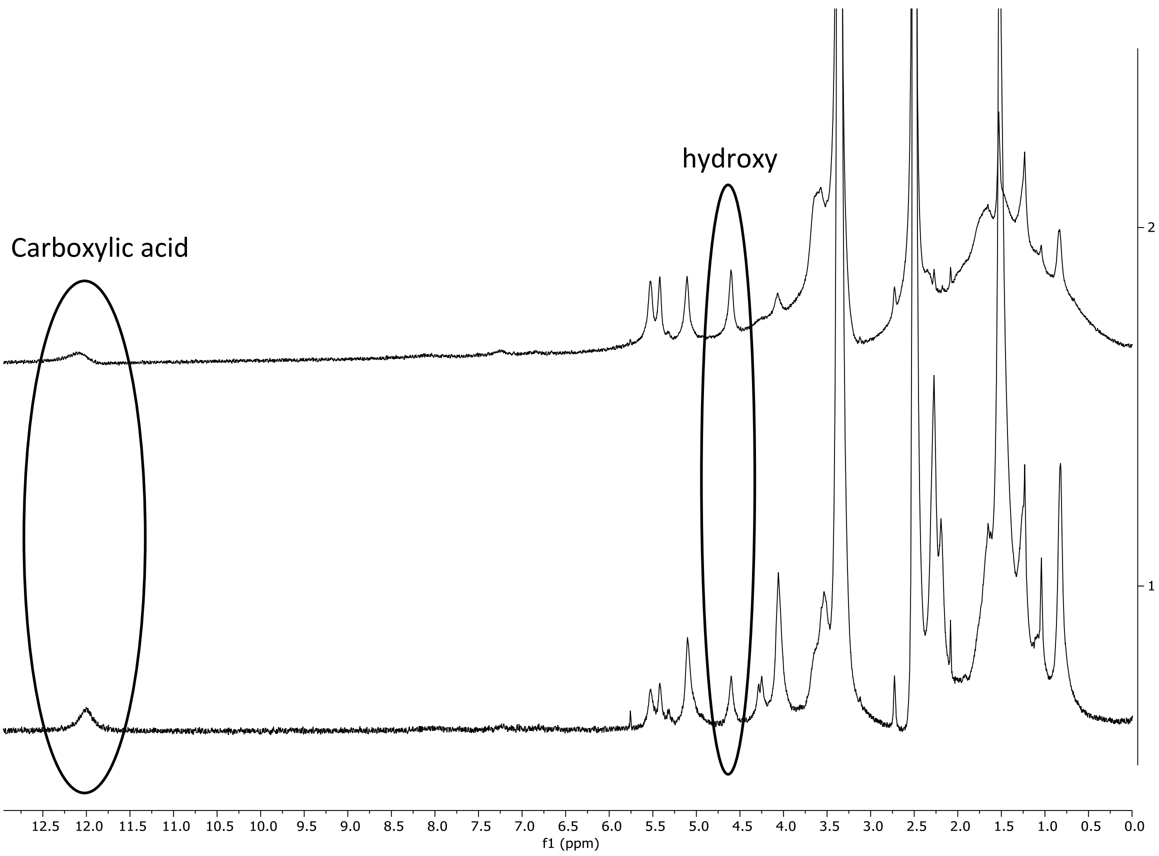

Figure S24. ^1H NMR (DMSO-d_6) spectra of the hydrolyzed products of P3 (below) and P4 (above) after enzymatic degradation

II. LC-MS

a. HBO-citro

$C_{15}H_{26}O_4$ (270.1831 g/mol)
271.1897 g/mol identified at 10 min
The theoretical mass of $[M+H]^+$ is 271.1904 g/mol

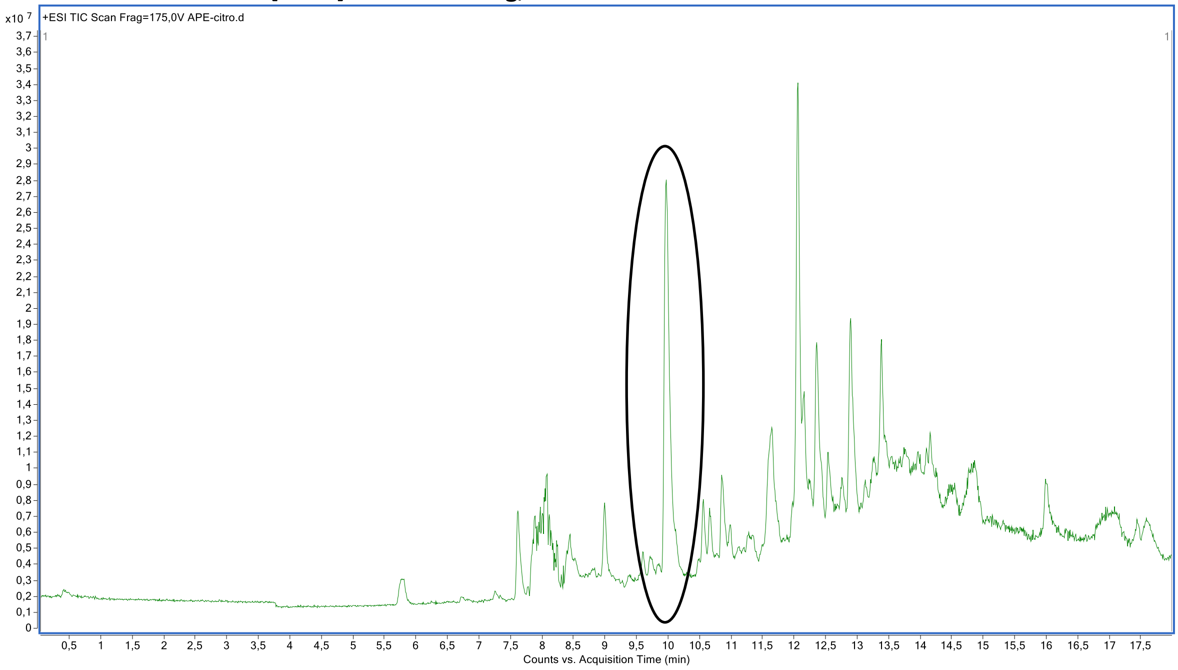

Figure S25. LC-MS chromatogram of HBO-citro

b. Triol-citro

$C_{15}H_{30}O_4$ (274.2144 g/mol)
275.2213 g/mol identified at 8.6 min
The theoretical mass of $[M+H]^+$ is 275.2217 g/mol

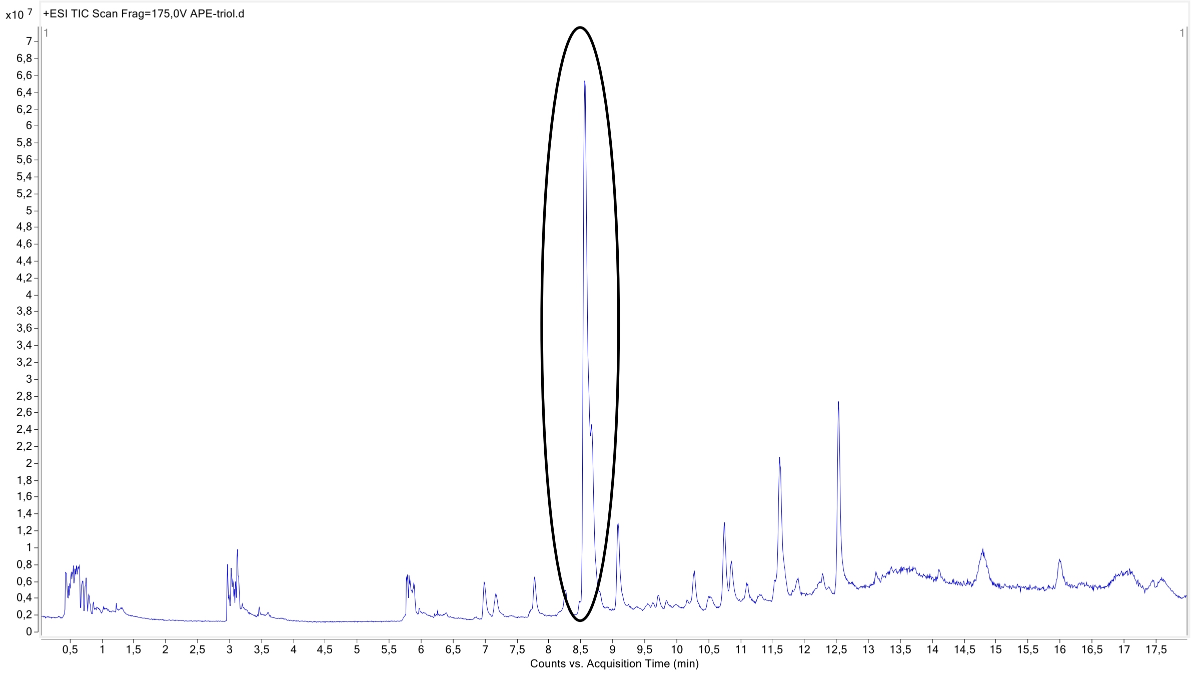

Figure S26. LC-MS chromatogram of Triol-citro

III. SEC

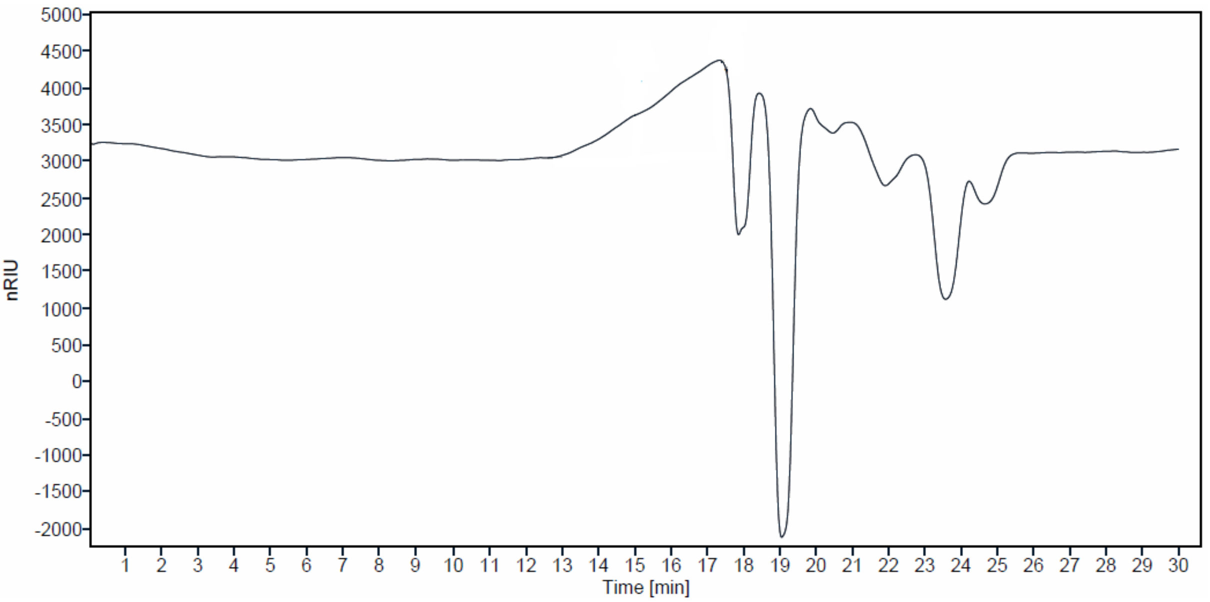

Figure S27. SEC trace of P1, Table 2

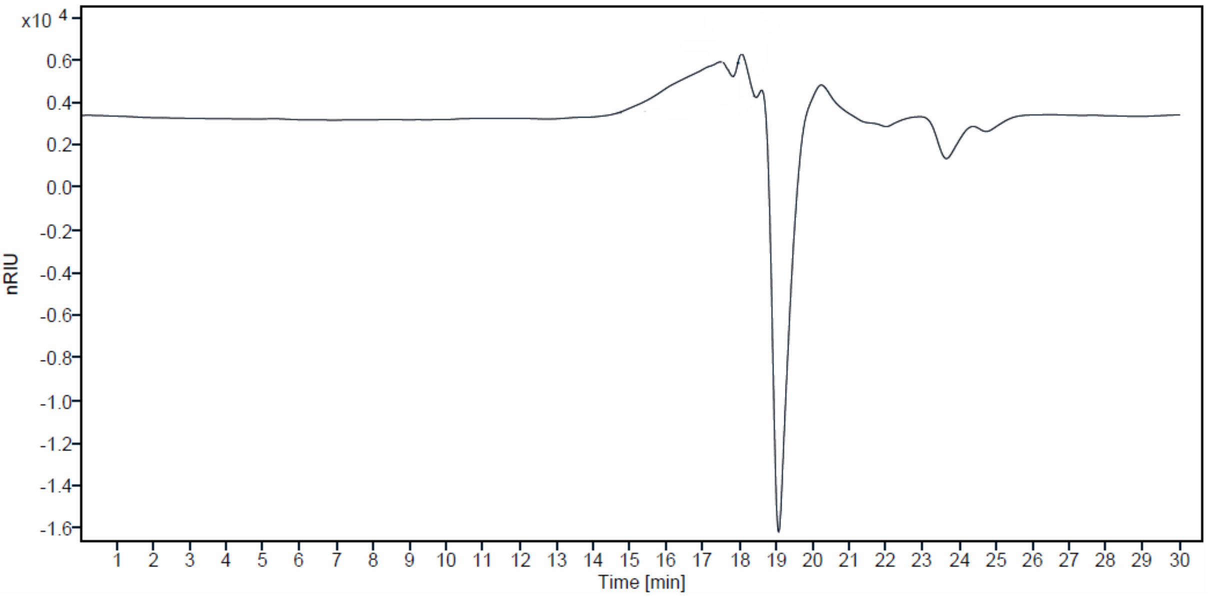

Figure S28. SEC trace of P2, Table 2

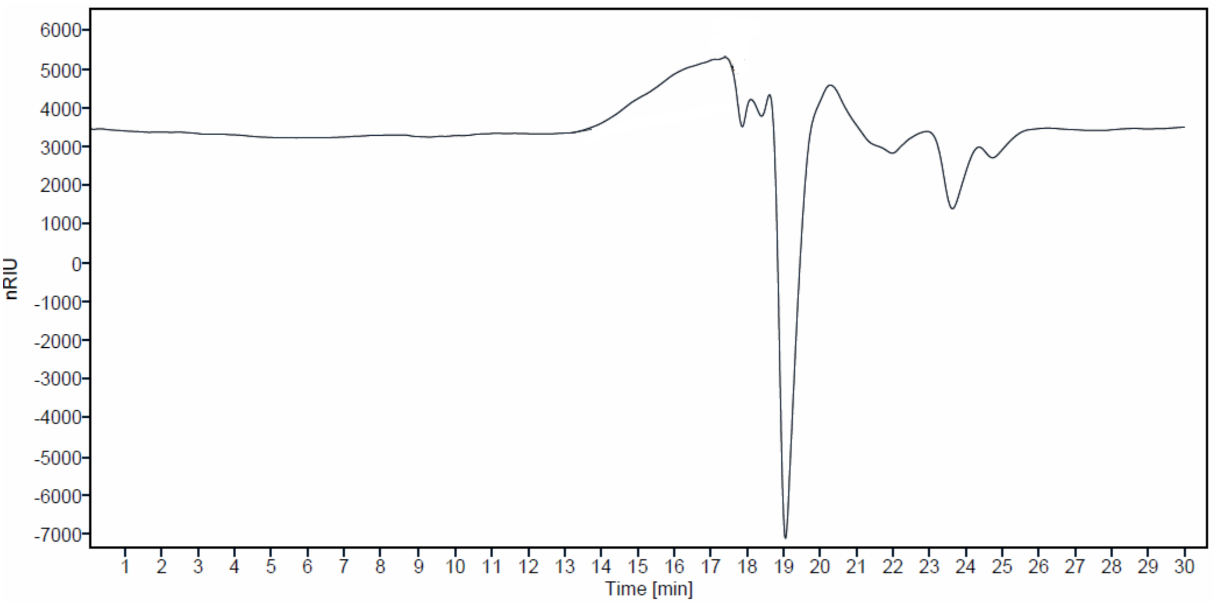

Figure S29. SEC trace of P3, Table 2

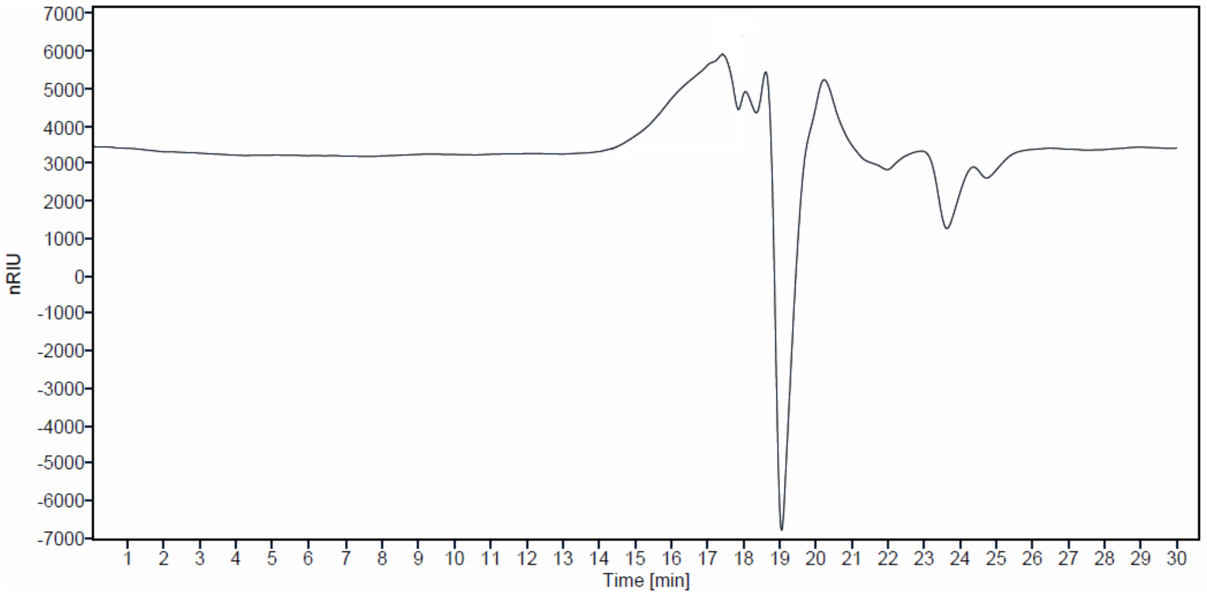

Figure S30. SEC trace of P4, Table 2

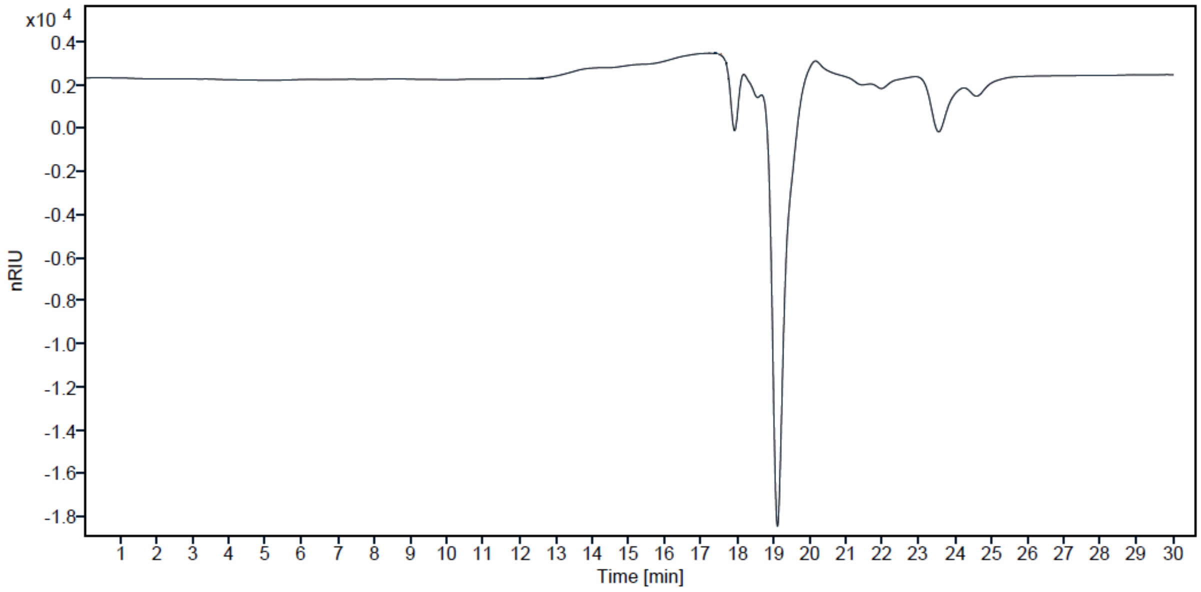

Figure S31. SEC trace of the resulting product of P1 (run 1, Table 2) after enzymatic degradation

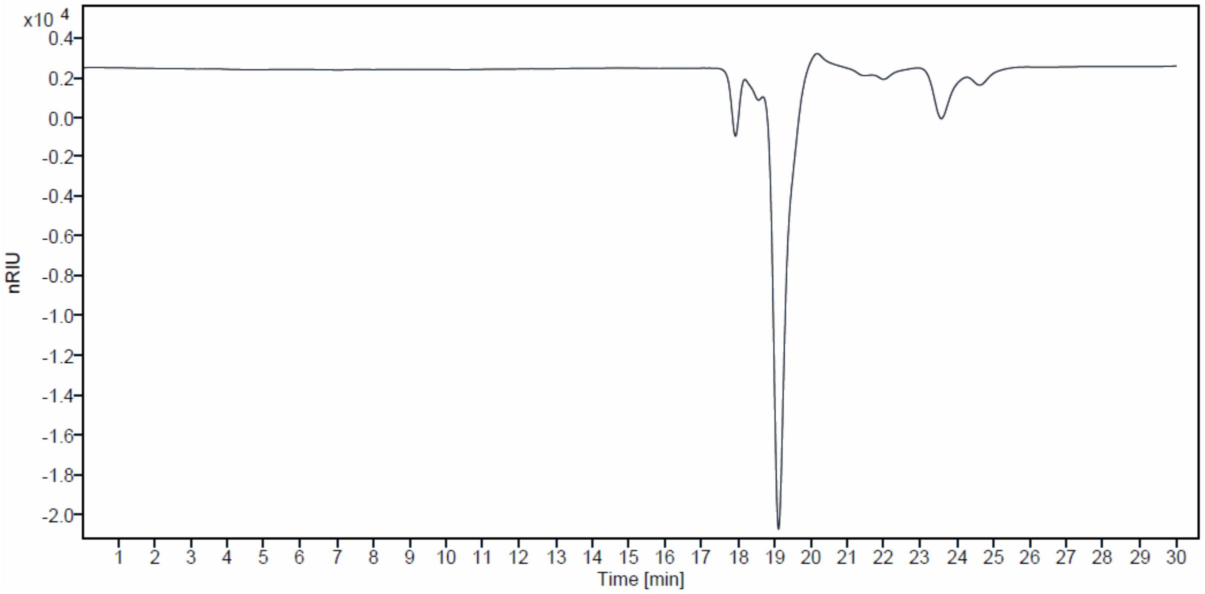

Figure S32. SEC trace of the resulting product of P2 (run 2, Table 2) after enzymatic degradation

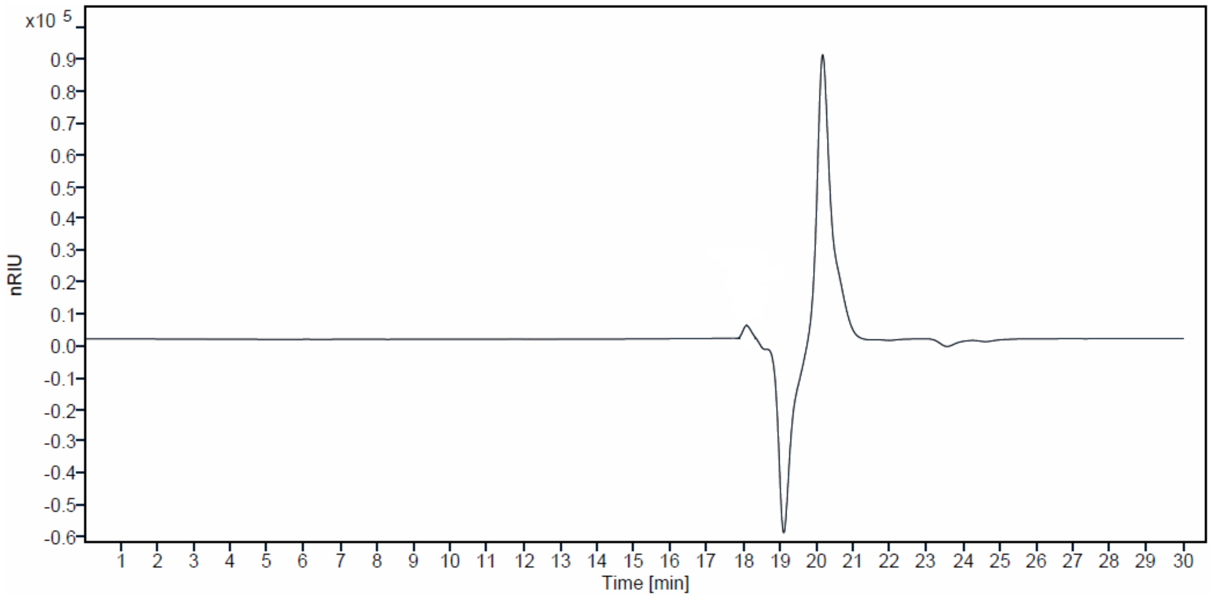

Figure S33. SEC trace of the resulting product of P3 (run 3, Table 2) after enzymatic degradation

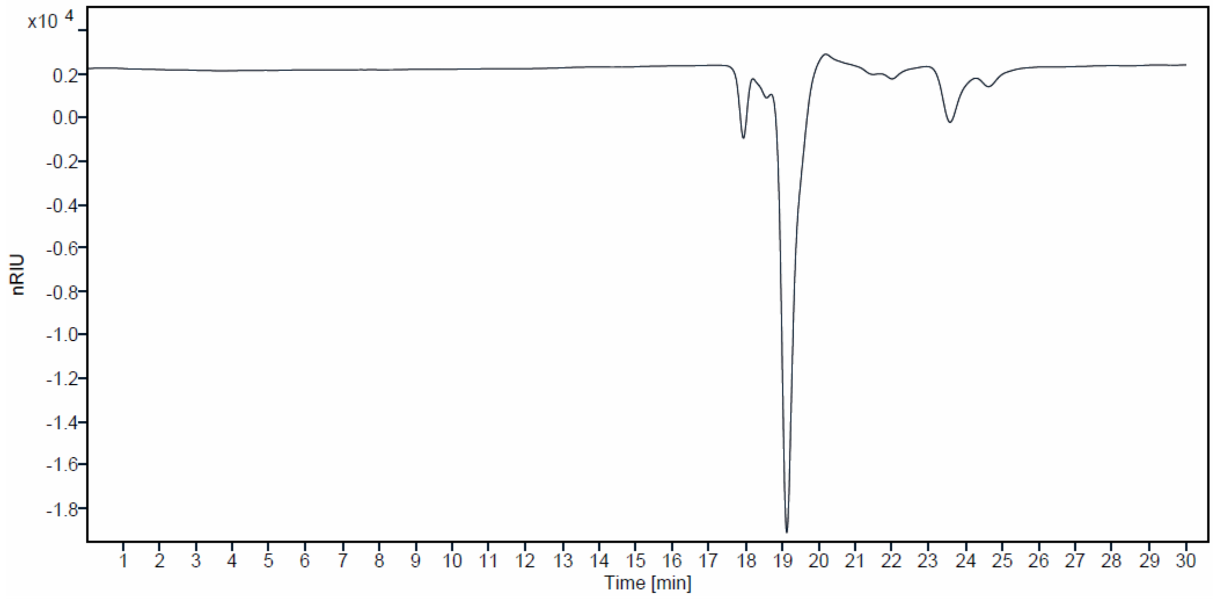

Figure S34. SEC trace of the resulting product of P4 (run 4, Table 2) after enzymatic degradation

IV. FTIR

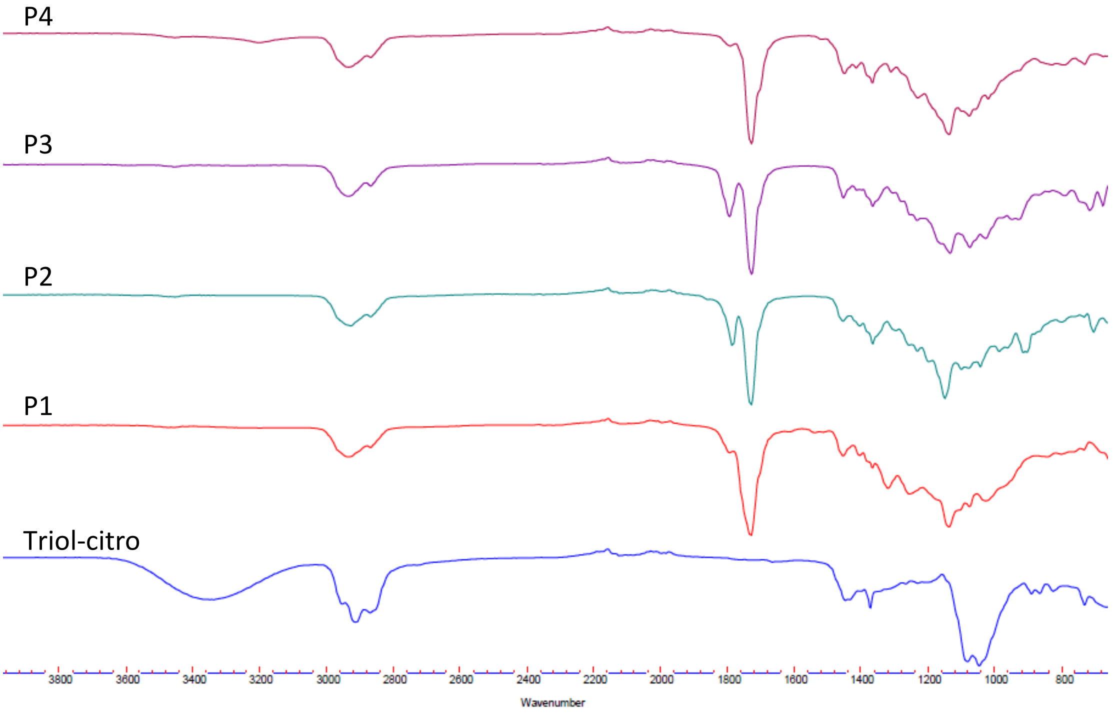

Figure S35. Typical FTIR spectra of P1-P4 (runs 1-4, Table 2)

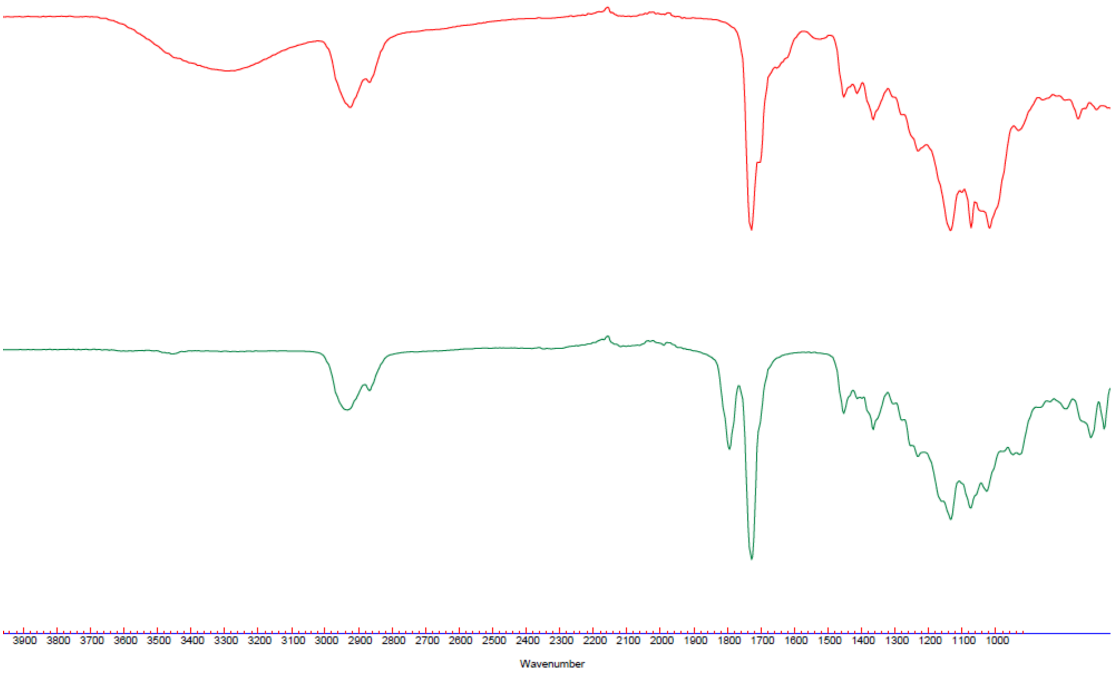

Figure S36. Typical FTIR spectra of P3 (taken as an example) before and after enzymatic degradation (before: green and after: red, respectively)

V. DSC

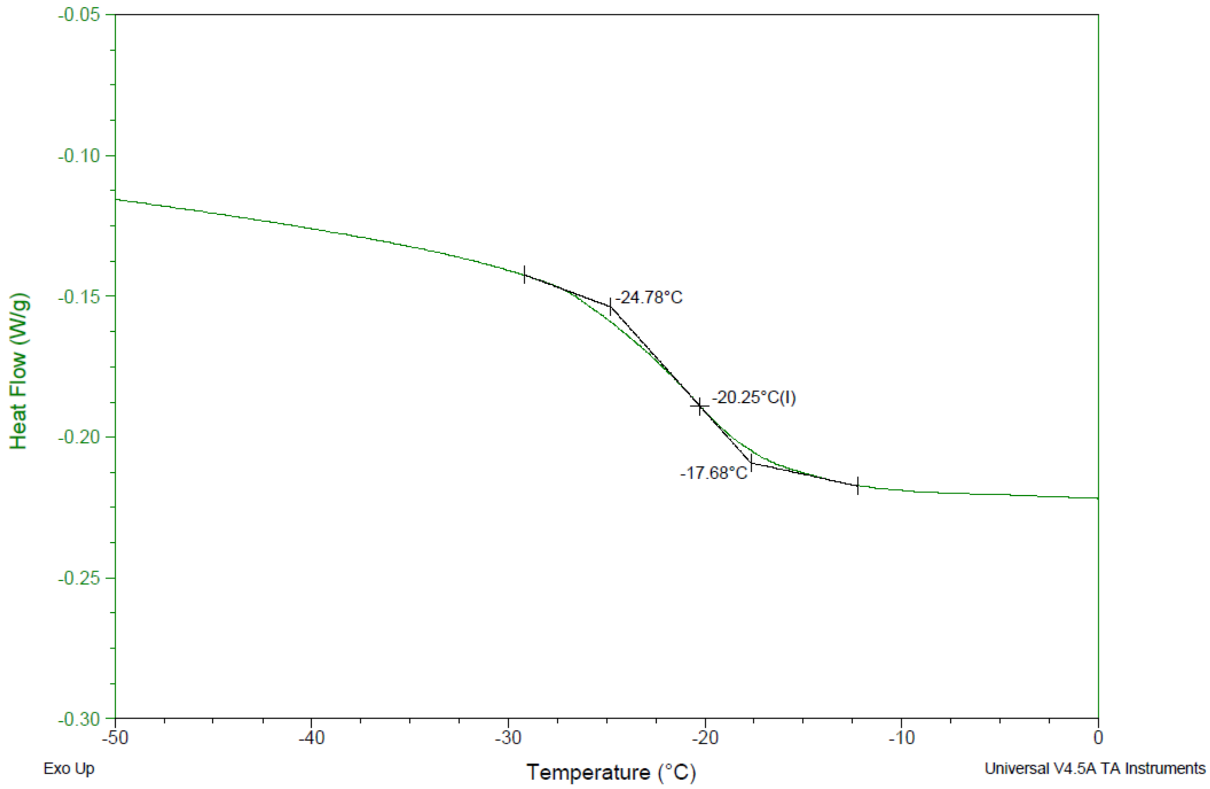

Figure S37. DSC thermogram (3rd heat cycle) of P1, run 1, Table 2

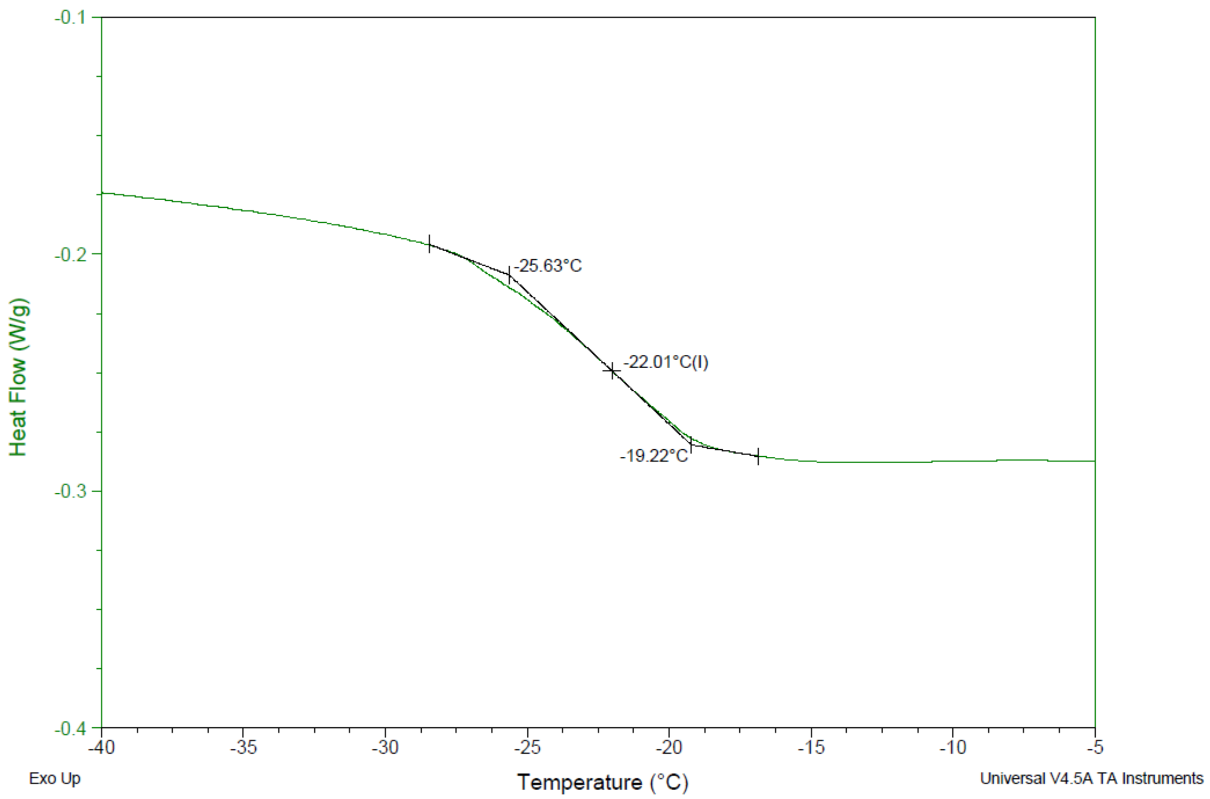

Figure S38. DSC thermogram (3rd heat cycle) of P2, run 2, Table 2

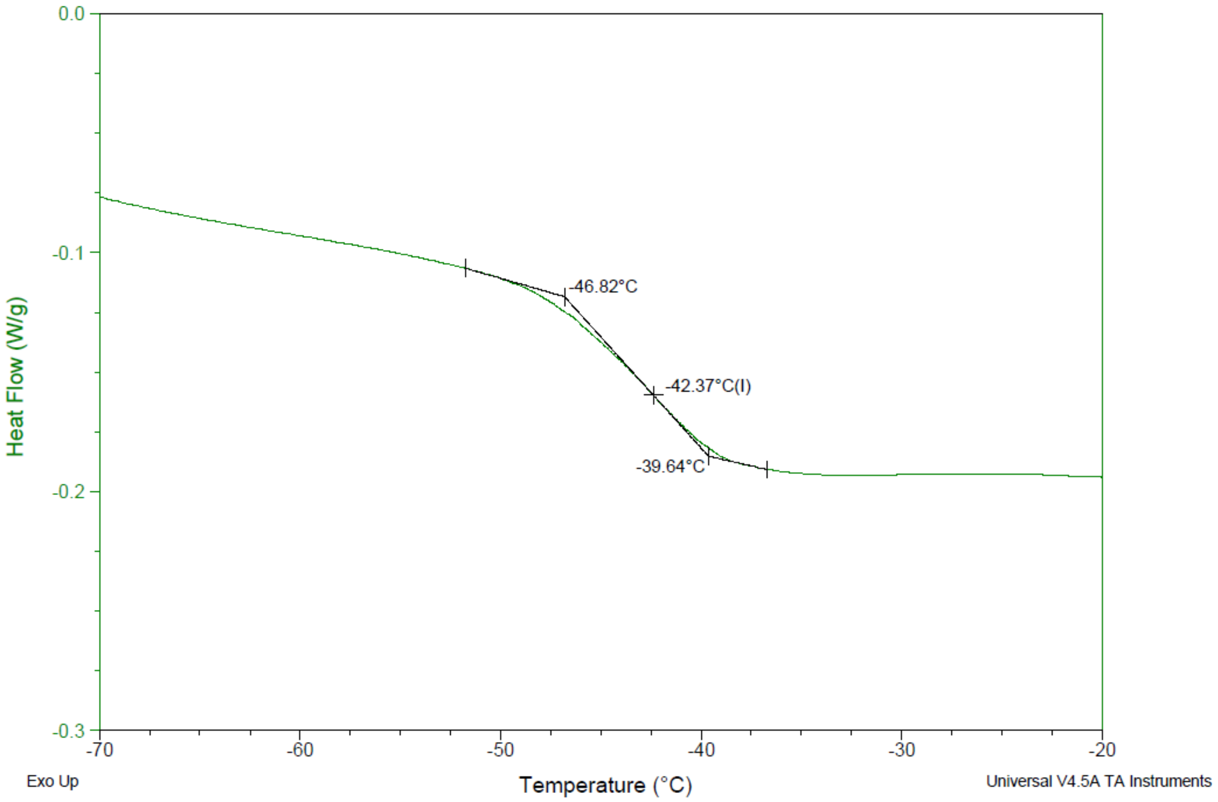

Figure S39. DSC thermogram (3rd heat cycle) of P3, run 3, Table 2

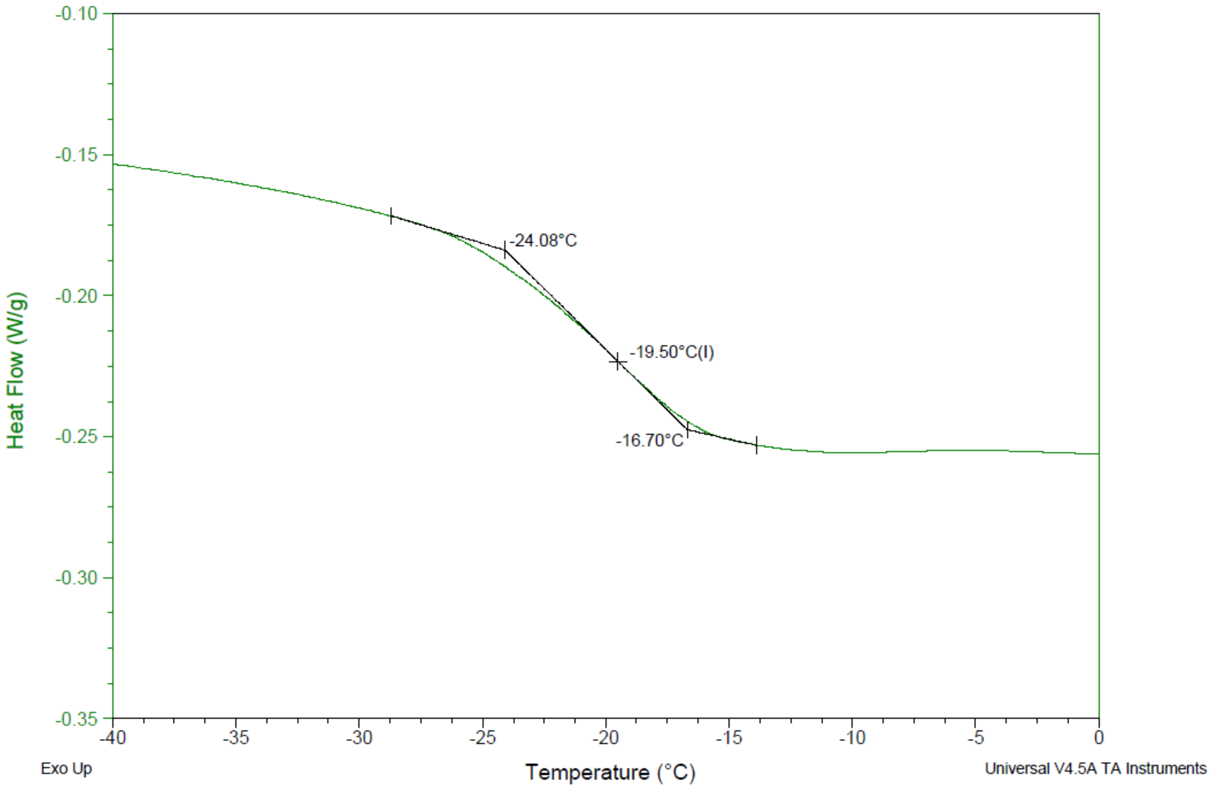

Figure S40. DSC thermogram (3rd heat cycle) of P4, run 4, Table 2

VI. TGA

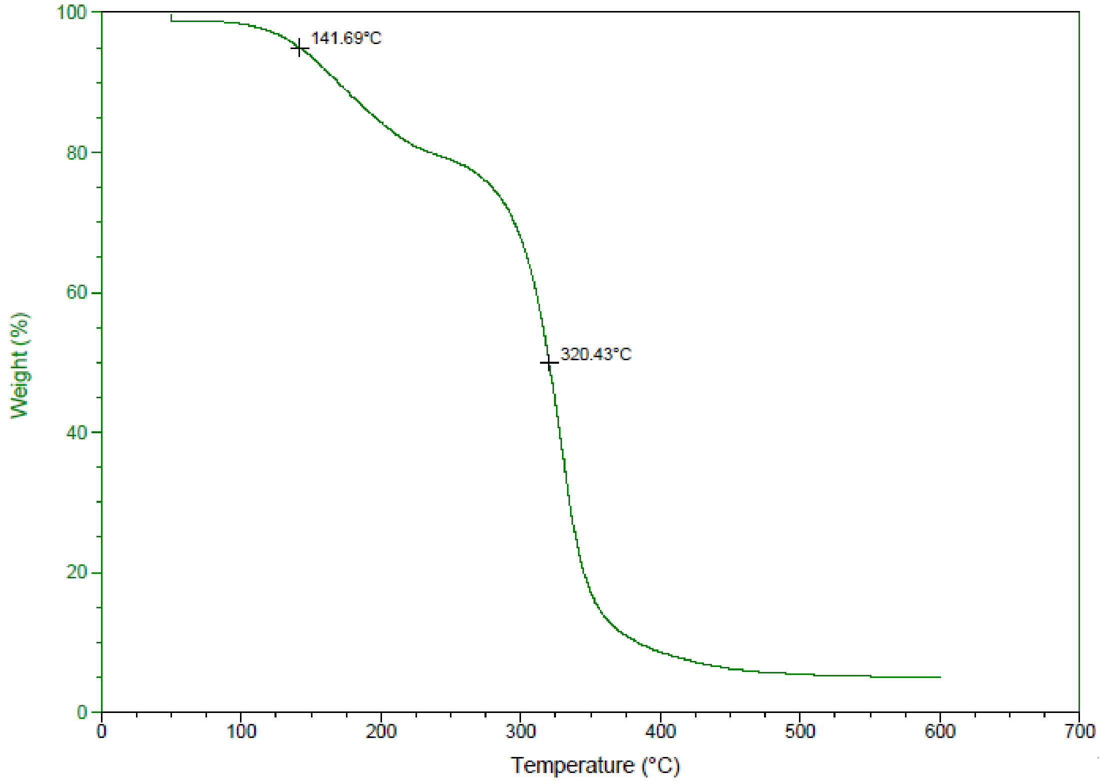

Figure S41. TGA thermogram of P1, run 1, Table 2

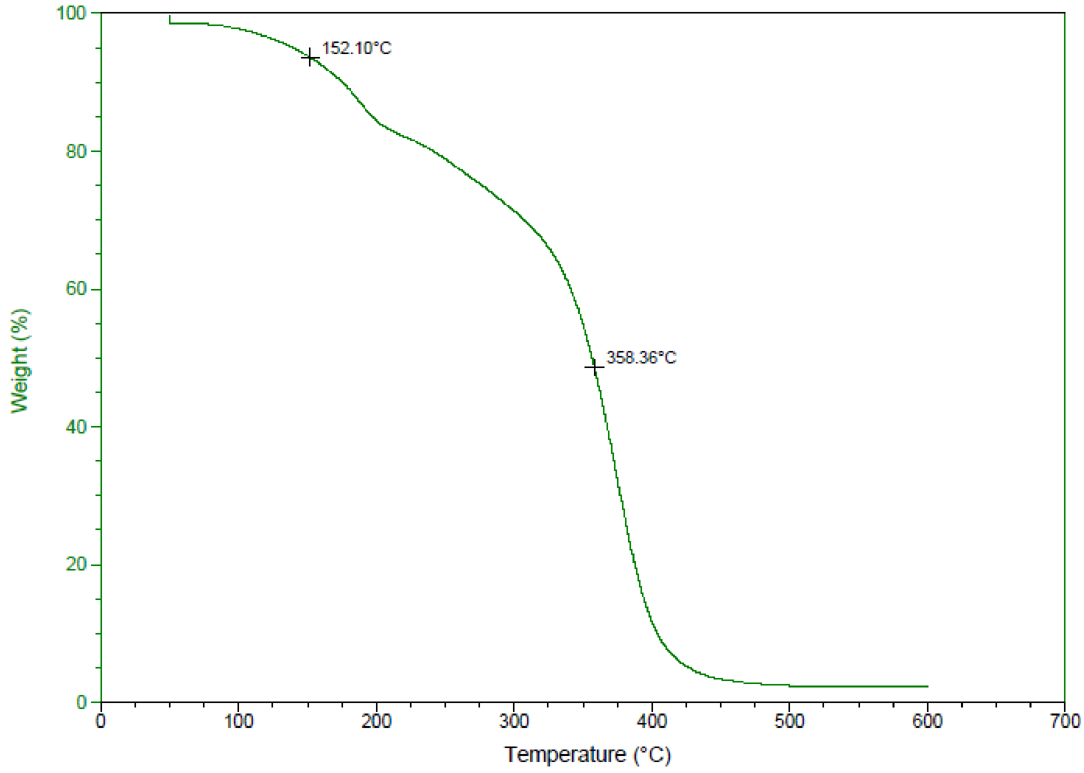

Figure S42. TGA thermogram of P2, run 2, Table 2

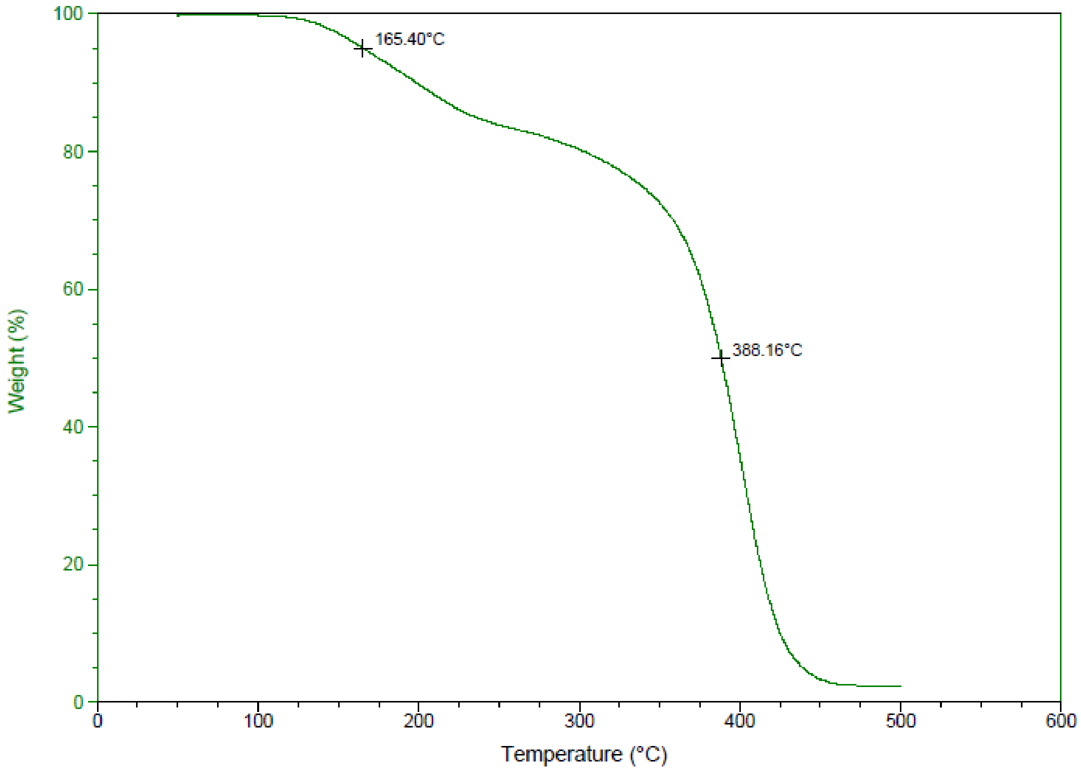

Figure S43. TGA thermogram of P3, run 3, Table 2

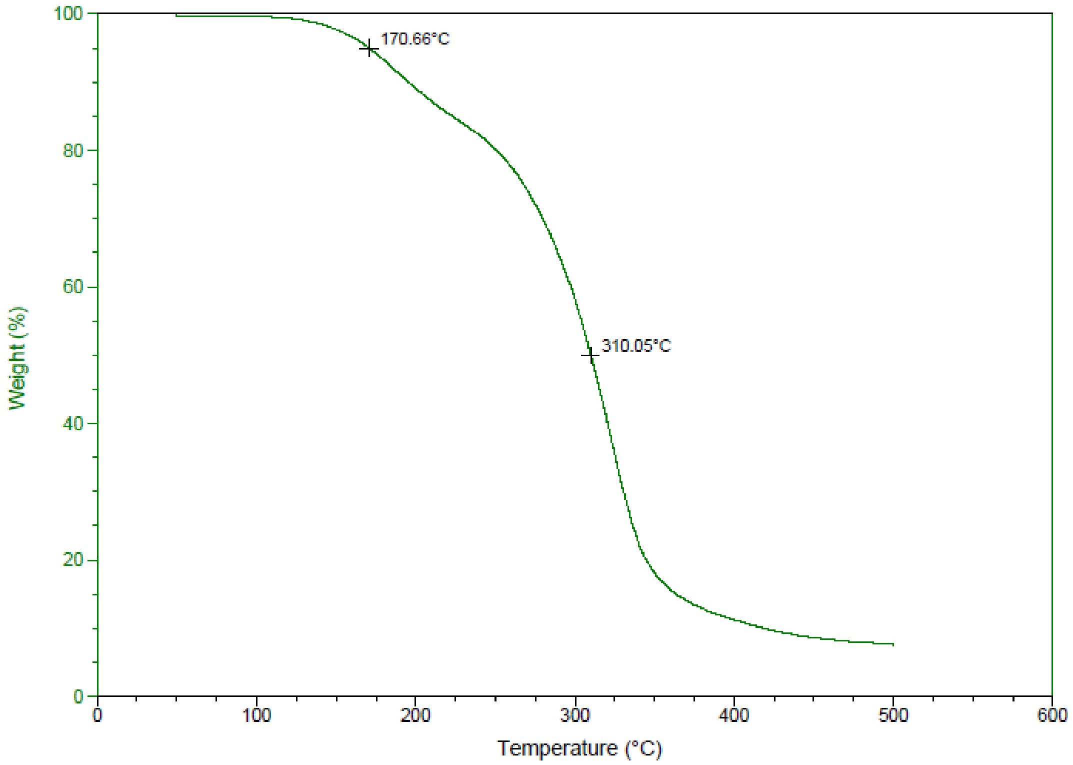

Figure S44. TGA thermogram of P4, run 4, Table 2
